# A systems biology approach identifies candidate drugs to reduce mortality in severely ill patients with COVID-19

**DOI:** 10.1126/sciadv.abm2510

**Published:** 2022-06-01

**Authors:** Vinicius M. Fava, Mathieu Bourgey, Pubudu M. Nawarathna, Marianna Orlova, Pauline Cassart, Donald C. Vinh, Matthew Pellan Cheng, Guillaume Bourque, Erwin Schurr, David Langlais

**Affiliations:** 1 Infectious Diseases and Immunity in Global Health Program, The Research Institute of the McGill University Health Centre, Montreal, QC, Canada.; 2 McGill International TB Centre, McGill University, Montreal, QC, Canada.; 3 Canadian Centre for Computational Genomics, McGill University, Montréal, QC, Canada.; 4 Department for Human Genetics, McGill University Genome Centre, McGill University, Montréal, QC, Canada.; 5 Division of Infectious Diseases and Division of Medical Microbiology, McGill University Health Center, McGill University, Montreal, QC, Canada.; 6 Department of Microbiology and Immunology, McGill University, Montreal, QC, Canada.; 7 McGill University Research Centre on Complex Traits, Montreal, QC, Canada.

## Abstract

Despite the availability of highly efficacious vaccines, coronavirus disease 2019 (COVID-19) caused by severe acute respiratory syndrome coronavirus 2 (SARS-CoV-2) lacks effective drug treatment, which results in a high rate of mortality. To address this therapeutic shortcoming, we applied a systems biology approach to the study of patients hospitalized with severe COVID. We show that, at the time of hospital admission, patients who were equivalent on the clinical ordinal scale displayed significant differential monocyte epigenetic and transcriptomic attributes between those who would survive and those who would succumb to COVID-19. We identified messenger RNA metabolism, RNA splicing, and interferon signaling pathways as key host responses overactivated by patients who would not survive. Those pathways are prime drug targets to reduce mortality of critically ill patients with COVID-19, leading us to identify tacrolimus, zotatifin, and nintedanib as three strong candidates for treatment of severely ill patients at the time of hospital admission.

## Teaser

Epigenetics distinguishes COVID-19 survivors already at hospital admission: lessons for drug repurposing.

## INTRODUCTION

Coronavirus disease 2019 (COVID-19) caused by severe acute respiratory syndrome coronavirus 2 (SARS-CoV-2) is characterized by heterogeneous clinical outcomes of infection ranging from absence of clinical symptoms to death. Intense research has resulted in the generation of highly effective vaccines for the prevention of COVID-19–related hospitalization and death (*1*–*5*). However, considering the ongoing circulation of the virus, the appearance of new variants with potential for escape of vaccine-induced immunity, the emergence of vaccine breakthroughs, and widespread vaccine hesitancy, treatment of severely symptomatic patients with COVID-19 remains a clinical challenge. Unfortunately, the success of these vaccines has not been paralleled by the development of efficacious drugs for the treatment of COVID-19, and there is an urgent need for effective therapies to treat severely ill patients with COVID-19. Treatment options include antiviral drugs (e.g., remdesivir), which have, so far, demonstrated only a discrete utility in the nonsevere setting, or host-directed therapies. Of the latter, dexamethasone is currently recommended for patients who require supplemental oxygen, yet mortality rates remain in excess of 20% among critically ill patients (*6*). Therapeutic anticoagulation may reduce mortality in those with mild or moderate disease (*7*), while anti–interleukin-6 antibodies (e.g., tocilizumab and sarilumab) can be added to steroid treatment for patients with severe manifestations and inflammation from SARS-CoV-2 (*8*). Remdesivir is currently approved by the Food and Drug Administration for treatment of patients with COVID-19 (*9*), yet it has not been shown to improve mortality in patients hospitalized with COVID-19 and is not recommended by the World Health Organization (WHO) (*10*). A clinical trial in Spain showed that cyclosporine reduced mortality in hospitalized patients with severe COVID-19 (*11*). Because the development of new antiviral drugs is a costly and time-consuming activity, drug repurposing has been the main avenue for discovery of treatment options for patients with COVID-19 (*12*, *13*).

Efforts of repurposing have focused on scanning key pathways of viral replication and host responses as possible drug targets. Protein-protein or protein-RNA interaction screens have not only provided exciting insight into the basic mechanisms of the SARS-CoV-2 life cycle but also identified a multitude of candidate drugs for possible COVID-19 therapy (*14*–*18*). Similarly, artificial intelligence and computational modeling have been used to identify candidate repurposed drugs (*19*–*21*). Because results of these studies strongly rely on cellular assays, it is difficult to know how these findings will translate to patients. Patient-centric approaches have been focused on high-resolution studies of immune processes and host responsiveness of hospitalized patients. These studies have defined key host responses that drive COVID-19 pathology and severity and have pinpointed the type I interferon response as a critical regulator of COVID-19 severity (*22*–*28*). In addition, these studies have identified single-cell features, cell types, and gene markers associated with the degree of COVID-19 pathology (*28*–*31*). However, these studies were primarily concerned with understanding COVID-19 pathophysiology and generally did not pursue a drug-repurposing strategy.

Given the spectrum of clinical COVID-19 presentations, it seems highly plausible that different drugs are needed for different disease stages. In our study, we have focused on critically ill patients for whom life-saving treatment remains an urgent unmet need. We reasoned that, by contrasting critically ill patients who recover with those who perish, we would be able to identify host response pathways segregating between these two outcomes. These pathways could then be pharmacologically targeted through enhancement- or suppression-based strategies. We enrolled patients with the same severe clinical WHO ordinal scale (OS = 7) at hospital admission and followed these patients for 15 days after admission. Of the seven enrolled patients, three died of COVID-19 while four recovered and were discharged. We contrasted the transcriptome and epigenetic landscape of immune cells of the two patient groups at admission as well as at days 5 and 15 after admission as a follow-up. We found that disease evolution and survival are mainly characterized by changes in cellular composition with relatively modest impact on the transcriptome of key effector cells at later stages of the disease. In contrast, at the time of hospital admission, we detected significant transcriptional changes in key molecular pathways that are associated with epigenetic changes in monocytes. Of importance for the objective of the present study, at a time when the patients were of the same clinical severity, we identified the spliceosome and RNA metabolism as main targets for repurposed drugs to be deployed at the earliest point of hospitalization of critically ill patients.

## RESULTS

### Multiomics profiling of severely ill hospitalized patients with COVID-19

**Fig. 1.**
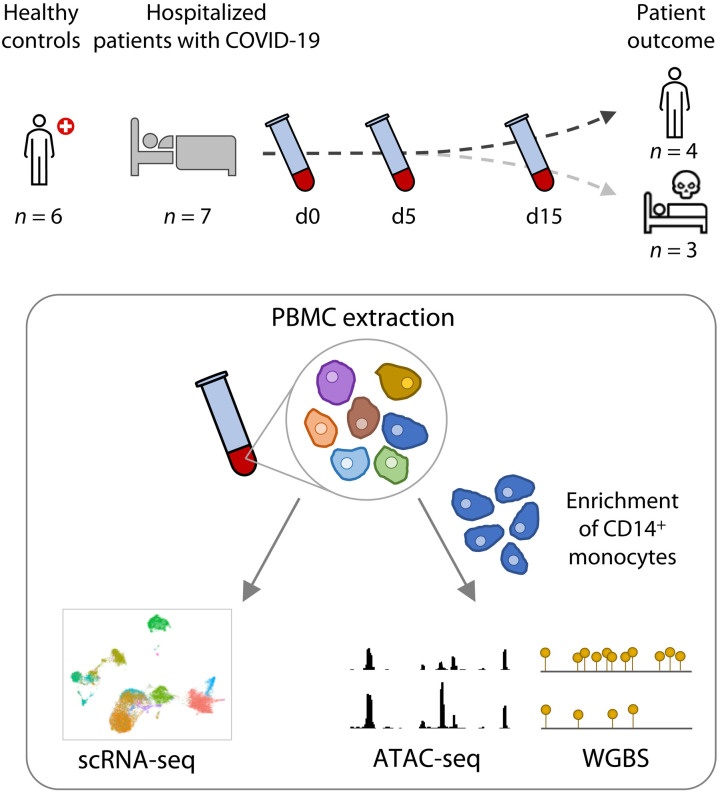
Study design summary. Blood samples were collected from healthy controls and from severely ill patients with COVID-19 at the time of hospitalization (d0) and at follow-ups (days 5 and 15) during their stay in the critical care unit. PBMCs were isolated from the blood and captured for single-cell RNA sequencing (scRNA-seq). CD14^+^ monocytes were also enriched from PBMCs for epigenetic analyses: chromatin accessibility by assay for transposase-accessible chromatin sequencing (ATAC-seq) and DNA methylation by whole-genome bisulfite sequencing (WGBS).

We enrolled seven treatment-naive hospitalized patients with COVID-19 (P1 to P7) at the McGill University Health Centre (MUHC), Montréal, Québec, Canada. Patients were aged from 31 to 92 years (Table 1). Among the seven patients, three were females and four were males. The average time from onset of symptoms to admission was 6 ± 4.5 (SD) days, and the average time from diagnosis to admission was 3 ± 3.2 days. At admission, all patients were classified with an OS of 7 in the WHO Clinical Progression Scale (*32*). Three patients (one female and two males, aged 31, 48, and 92) succumbed to the disease at days 8, 16, and 35 after hospital admission. For all patients, peripheral blood mononuclear cells (PBMCs) were obtained by phlebotomy followed by standard density gradient centrifugation at three time points: at admission as well as 5 and 15 days after recruitment (for surviving patients). This design is unique in comparison to previously published single-cell analyses of PBMCs from patients with COVID-19, as it captured the patient on the day they were reaching an OS of 7 and requiring intensive care unit admission. This allowed us to follow disease evolution from the same clinical reference point and to identify the epigenomics and cellular factors associated with COVID-19 disease evolution (Fig. 1). Of importance, the retrospective classification of patients at admission in survivors and nonsurvivors permitted us to identify early changes in the overall immune status as high-value targets for pharmacological intervention to block poor outcomes including death.

**Table 1.** Patient demographics. HTN, hypertension; no PMHx, no previous medical history; Hep C, hepatitis C; BIPAP, bilevel positive airway pressure; PEG, percutaneous endoscopic gastrostomy; DLP, dyslipidemia; DM, diabetes mellitus; BPH, benign prostatic hyperplasia; Sx, symptoms; N/A, not applicable; SOC, standard of care.

**Participant**	**Sex**	**Age**	**Comorbidities**	**Ethnicity**	**Date of** **Sx onset**	**Date of** **diagnosis**	**Date of** **admission**	**COVID-19** **treatment**	**OS**					**Days** **hospitalized**	**Status at** **discharge**
									**Day 0**	**Day 3**	**Day 5**	**Day 8**	**Day 15**		
P1	F	46	HTN and anemia	Asian	26 March 2020	8 April 2020	9 April 2020	SOC	7	7	6	7	4	7	Alive
P2	M	49	No PMHx	Latin American	16 April 2020	10 April 2020	16 April 2020	SOC	7	7	8	5	4	27	Alive
P3	F	31	HTN	White	N/A	11 April 2020	18 April 2020	Kaletra	7	9	8	DCD	DCD	9	Deceased
P4	M	48	Hep C and renal disease	Black	9 April 2020	16 April 2020	16 April 2020	SOC	7	9	9	8	9	35	Deceased
P5	M	41	Quadriplegic, continuous BIPAP; neurogenic bladder; and PEG	White	7 April 2020	18 April 2020	18 April 2020	SOC	7	4	6	4	4	30	Alive
P6	F	78	Osteoporosis, DLP, osteoarthritis, and right total hip arthroplasty	White	30 April 2020	30 April 2020	7 May 2020	SOC	7	5	4	4	4	22	Alive
P7	M	92	Mild cognitive impairment, HTN, DM type 2, BPH, and spinal stenosis	White	17 May 2020	17 May 2020	18 May 2020	SOC	7	6	6	6	6	16	Deceased

### Change in cellular components is associated with disease severity

**Fig. 2.**
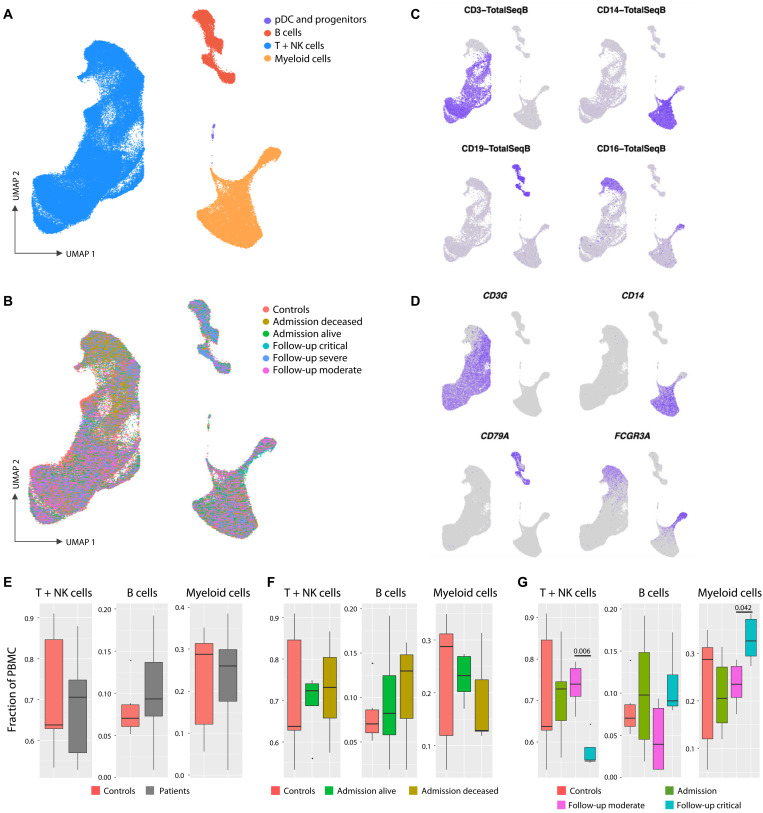
Cellular composition of PBMCs from severely ill hospitalized patients with COVID-19 and controls. (**A**) UMAP visualization of PBMCs from seven patients with COVID-19 (18 samples obtained at days 0, 5, and 15 of admission) and six healthy controls identified four major PBMC lineages. (**B**) Cells are colored according to the clinical classification of patients with COVID-19 and healthy controls. (**C**) Visualization of four cell surface lineage–specific markers across cell clusters using TotalSeqB antibodies on control samples reveals T cells (CD3), CD14^+^ monocytes (CD14), B cells (CD19), and CD16^+^ and NK cells (CD16). (**D**) Cells are colored according to the expression of the lineage marker genes *CD3G* (T cells), *CD14* (CD14^+^ monocytes), *CD79A* (B cells), and *FCGR3A* (CD16^+^ monocytes and NK cells). (**E** to **G**) Box-and-whisker plots for the proportion of cells in each major lineage compared to the overall number of cells in the sample for T + NK, B, and myeloid cells among clinical groups. The PBMC lineage proportions are shown for healthy control samples (red) versus (E) all COVID-19 samples (gray), (F) samples collected from patients with COVID-19 at admission and retrospectively classified as deceased (brown) or alive (green), and (G) samples at days 5 and 15 for patients classified as displaying moderate (magenta) or critical (cyan) clinical symptoms. *q* values for pairwise *t* test comparisons (Admission - deceased versus alive; Follow-up - critical versus moderate) are provided when significant.

We used a single-cell RNA sequencing (scRNA-seq) approach to understand the cellular composition and transcriptional status of PBMCs following hospitalization and compared the results with those obtained for six healthy controls. Following quality filtering and doublet removal, a total of 105,851 cells were integrated and then clustered using a uniform manifold approximation and projection (UMAP) analysis. All samples were first regrouped into three major PBMC populations, namely, B cells, myeloid cells, and T + natural killer (NK) lymphocytes (Fig. 2A). Cells from patients with COVID-19 and cells from six healthy controls were homogeneously represented among the three clusters (Fig. 2B). Clustering was confirmed by the expression of cell-specific surface antigens (Fig. 2C) and expression of known cell lineage marker genes (Fig. 2D). We ran subclustering analysis for each of the three main lineages to confidently identify subpopulations. The T + NK lineage could be divided into 12 subclusters (fig. S1), the B cells cluster into 4 subclusters (fig. S2), and the myeloid cell cluster into 7 subclusters (fig. S3), which also included the plasmacytoid dendritic cells (pDCs) and some hematopoietic stem and progenitor cells. Cell type–specific gene markers were extracted for every subcluster to identify the corresponding cell population.

PBMC lineage proportions were compared between patients and controls, as well as classes of patients (Fig. 2, E to G). In the global comparison, we did not detect significant differences in proportions of T cells, B cells, and myeloid cells between healthy controls and all severe COVID-19 samples (Fig. 2E). Similarly, when patients were retrospectively grouped according to subsequent recovery (“alive”) or death (“deceased”), we failed to detect significant differences in PBMC lineage proportion for the corresponding day 0 samples (Fig. 2F). In contrast, we observed a significant change in major lineage proportions when we pooled all samples according to their clinical status (i.e., critical versus moderate according to the OS score) at follow-up (day 5 and day 15; Fig. 2G). This suggested a strong impact of developing COVID-19 severity on PBMC proportions. Critically ill patients showed a significant reduction of T cells (*P* = 0.006) and a significant increase of myeloid cells (*P* = 0.04). These are accounted for by the changes in specific cellular subsets, where (i) the decrease in T cell numbers was driven by a specific reduction of naive (*P* = 0.004) and central memory (T_cm_; *P* = 0.04) CD4^+^ T cell populations (fig. S4A), (ii) B cell subpopulations (fig. S4B) remained unchanged and confirmed the absence of association with disease severity, and (iii) the increase in myeloid cells was driven by a specific expansion of CD16^+^ monocytes (*P* = 0.03; fig. S4C).

### Transcriptional expression profile as a marker of the COVID-19 prognosis

To gain further insight in the molecular pathogenesis of COVID-19 severity, differential gene expression (DGE) analyses were conducted for PBMC populations. We excluded the pDC, cDC2 (conventional dendritic cells 2), and progenitor populations from this analysis because the total number of identified cells per population was too low (<300 absolute number of cells) to generate confident results. For comparisons, we used the same grouping as used for the analysis of lineage proportions, i.e., patients with COVID-19 “deceased versus alive” at day 0 and “critical versus moderate” at follow-up (see table S1 for detailed DGE results per lineage). All significantly differentially expressed genes (DEGs) (adjusted *P* value ≤ 0.05) irrespectively of their fold change (FC) were used for gene ontology (GO) enrichment analysis with the overexpressed and lower-expressed genes analyzed separately. Notably, including a log_2_FC cutoff of 0.5 did not affect the list of significant GO terms but might have reduced the quality of the module score analysis.

**Fig. 3.**
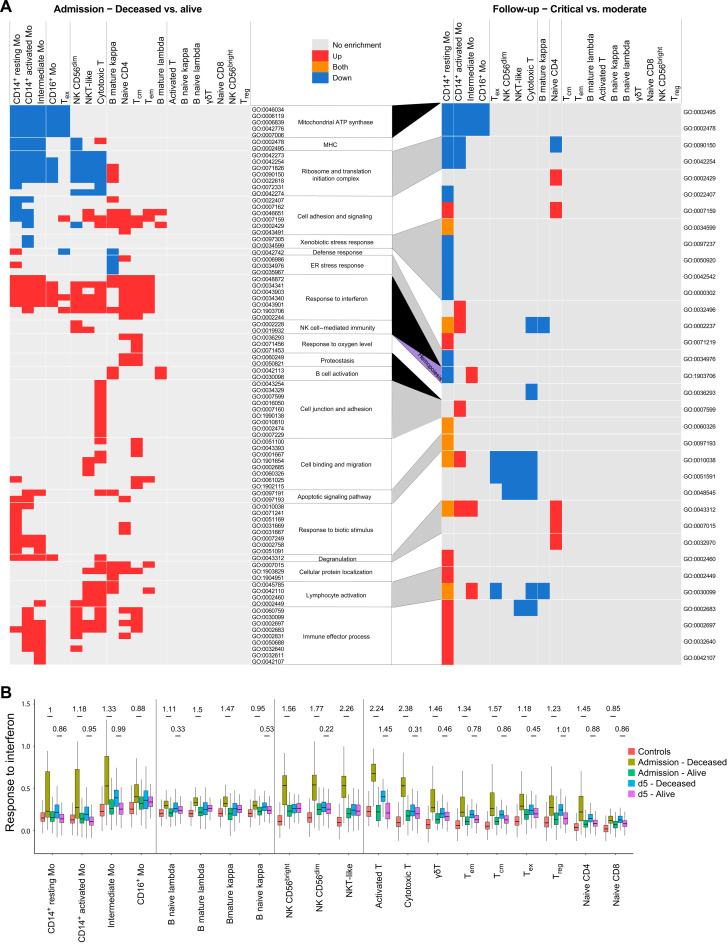
Transcriptional variations in cell populations of severely ill patients with COVID-19. (**A**) GO term enrichment analysis for DEGs at admission between the deceased versus alive patient groups (left heatmap) and between the critical versus moderate patients with COVID-19 at follow-up (right heatmap). Heatmap boxes are colored on the basis of the enrichment: gray for no significant enrichment; blue, red, and orange for significant (*q* value ≤ 0.05) enrichment of down-regulated, up-regulated, and both up- and down-regulated genes, respectively. GO categories only enriched at admission are highlighted in black between the two heatmaps, whereas the GO category highlighted in yellow (hemopoiesis) represents the only category enriched only at follow-up. GO categories enriched at both admission and follow-up are successively highlighted in white and gray for easier visualization. (**B**) Evolution of the transcriptional expression for the “response to interferon” GO term. DEGs enriched in the GO term in (A) were used to generate a module score representing the overall expression level for term genes in the cell lineage indicated at the bottom of the graph. Module scores shown were derived at admission and at the day 5 time points for the deceased and alive patients with COVID-19 separately. Control samples from healthy participants are included as reference. Significance of differences between deceased and alive patients with COVID-19 was tested by comparing the corresponding module score distribution in every cell subset using a Wilcoxon rank test approach (*q* value ≤ 0.05). To provide a sample-size free evaluation of the difference in expression, Cohen’s *d* effect size estimation is shown for every significant variation. Transcriptional evolution graphs for the other enriched GO categories are provided in fig. S5. T_em_, effector memory T cells; T_ex_, exhausted T cells; T_reg_, regulatory T cells; gdT, γδ T cells; B cells, either naive or memory cells expressing either the κ or λ light chain.

The significantly enriched GO terms (adjusted *P* value ≤ 1 × 10^−5^ and enriched for at least five DEGs) in at least one cell population were selected and reexamined in every population for the above contrasts. The corresponding results are summarized in the heatmap in Fig. 3A and detailed in table S2. Notably, despite no differences in WHO clinical score at admission, we observed more transcriptional changes and thus more enriched pathways for the decreased versus alive comparison at day 0 than for the critical versus moderate contrast at later time points (Fig. 3A).

For example, the “response to interferon” pathway is enriched in the deceased patients at admission, but this difference attenuated at later time points, similarly with other pathways related to response to infections and immune activation processes, as well as cell junction, adhesion, and migration. Other pathways related to mitochondrial adenosine triphosphate (ATP) synthase, major histocompatibility complex (MHC), ribosomes, and translational initiation are less expressed on day 0 in cells from deceased patients. On the basis of GO enrichment at admission, we identified three groups of cell populations: high responder cells (HR-cells: CD14^+^ and intermediate monocytes), moderate responder cells [MR-cells: CD16^+^ monocytes, mature B cells, cytotoxic NK CD56^dim^, and various T cell subsets: NKT-like, cytotoxic, exhausted (T_ex_), T_cm_, effector memory (T_em_), and naive CD4^+^ T cells], and low responder cells [LR-cells: NK CD56^bright^ considered to be high cytokine producers, activated T cells, ɣδT, regulatory T cells (T_reg_), naive T CD8^+^, and naive B cells] with no significant pathway enrichment in the latter cells (Fig. 3A, top of heatmap).

To further investigate expression changes of genes enriched in GO terms and to depict the magnitude of the effect sizes, we considered enriched genes as modules and calculated module scores for each cell population to facilitate comparisons between participant groups. Module scores compute the average expression level of genes encompassed in each GO term and split by cell population and phenotype. Module scores were also computed for the deceased versus alive groups at day 5 to observe how these transcriptional changes progress along the disease evolution. At admission, the highest module score was obtained for genes in “response to interferon,” identifying this as the most up-regulated term, but only in patients that went on to die from COVID-19 (Fig. 3B), and this was observed not only for monocytes but also in NK and T lymphocytes. In both HR- and MR-cells, this was accompanied by the increased expression of genes involved in “immune effector” processes, suggesting that exacerbated immune responses and type I interferon signaling in HR- and MR-cells were linked with poor prognosis for patients (fig. S5I).

Overall, the module scores highlighted that activated/cytotoxic T cells and NK cells at admission are globally more activated than other lymphocyte populations in patients with poor prognosis, with strong induction of genes in the “immune effector process,” “NK cell–mediated immunity,” and “lymphocyte activation” terms (fig. S5). Similarly, T and NK cells exhibited increased expression of genes enriched in “cell junction and adhesion” and “cell binding and migration” terms. In addition, CD4^+^ naive T cells and T_cm_ cells displayed strong expression of genes enriched in “apoptosis,” “response to oxygen levels,” “proteostasis,” and “ER stress response” terms. Both naïve CD4^+^ and T_cm_ subpopulations underwent a strong reduction in numbers by days 5 and 15 after admission (fig. S4). In monocytes, poor prognosis at admission was also linked with a reduced expression of genes involved in the “mitochondrial ATP synthase” (mainly genes of the complex V), the “MHC,” and “ribosome and translation initiation” terms. Last, in mature B cells, we observed increased expression of genes enriched in the “B cell activation” term in nonsurvivors, consistent with a targeted response to SARS-CoV-2 in specific lymphocyte subpopulations.

We next compared the gene expression between patients who exhibited critical versus moderate symptoms at follow-up. Overall, transcriptional differences were small and correlated with the deceased prognosis at admission. Most of the terms that had shown significant enrichment at admission between deceased versus alive patients were not detected in this comparison (Fig. 3A). However, we still detected significant differences between gene expression module scores on day 5 especially in “response to interferon,” “immune effector process,” and “lymphocyte activation” terms (Fig. 3B and fig. S5). We noticed that monocytes expressed most of the remaining transcriptional differences between admission and the later stages of the disease. In addition, the module score analysis of deceased versus alive patients at follow-up showed lower or null effect size when compared to those observed at admission, consistent with the suggestion of a reduced transcriptional impact at later stages of the COVID-19 disease.

The reduced transcriptional differences contrasted with the substantial differences of PBMC lineage proportions that were only observed at later stages of hospitalization. This suggested that variations in transcriptional activity, and the accompanying epigenomics changes, mostly occurred at an early stage of COVID-19 disease, dictating how the disease will evolve in terms of severity and final outcome.

### The epigenetic profile of CD14^+^ monocytes correlates with poor prognosis for COVID-19

Given that monocytes had the largest number of transcriptional differences in our scRNA-seq, we sought to analyze their chromatin accessibility using the assay for transposase-accessible chromatin sequencing (ATAC-seq) on CD14^+^ monocytes enriched from PBMCs of hospitalized patients with COVID-19 over the 2-week follow-up (Fig. 1). We observed an increase in the fraction of reads in peaks (FRIP), a surrogate of cell quality and genomic stability, between day 0 and days 5 and 15 (fig. S6A). Genomic stability negatively correlated with an increase of the clinical OS, with critically ill cases presenting lower FRIP (fig. S6B). This correlation was not driven by differences in the fraction of CD14^+^ subsets. The high background resulting from the chromatin instability in patient monocytes restricted the comparison of genomic regions between phenotypic groups. Therefore, we tested for differential accessible chromatin (DAC) in deceased versus alive at admission and at follow-up for a total of 13,398 genomic regions where the FRIP surpassed the background at false discovery rate (FDR) < 1%. DAC was defined as genomic regions showing absolute log_2_FC > 0.5 and FDR < 5% between deceased versus alive at admission and at the follow-up. Because of the high background of the chromatin accessibility data, they should be interpreted in combination with DNA methylation and transcriptomic changes.

**Fig. 4.**
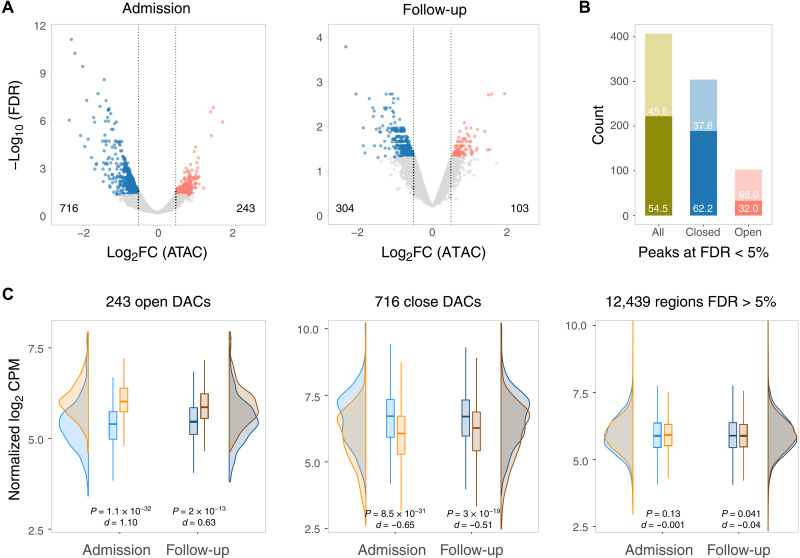
Monocyte chromatin accessibility among patients with COVID-19. (**A**) A total of 13,398 genomic regions identified as accessible by ATAC-seq were tested for DAC and plotted according to their log_2_FC and corresponding adjusted *P* value for the deceased versus alive comparison at day 0 and follow-up (days 5 and 15). More closed and more open chromatin regions in deceased patients at an FDR < 5% and absolute log_2_FC > 0.5 are shown in blue and red, respectively. (**B**) Intersection of DAC regions at admission and follow-up for the deceased versus alive group comparisons. The proportions of the 407 significant DAC regions at follow-up that were also significant at d0 are shown in the darker shade. The overlap for open DAC is indicated by the red bar, while the overlap for closed DAC is indicated by the blue bar. (**C**) Plots indicating the distribution of chromatin states for regions significantly more open (left), more closed (center), and not significantly different at FDR > 5% (right) for the deceased versus alive comparisons. The normalized log_2_ count per million (CPM) corresponding to accessibility levels for each of the chromatin regions denoted at the top was plotted in the *y* axis and summarized as density and box plots for the admission and follow-up samples. Blue shading indicates the chromatin state for the alive group, and orange shading indicates the deceased group. A Cohen’s *d* value was used to estimate the effect size, and a Wilcoxon test was used to assess the significance of the differences of the distributions between the deceased and alive patient samples.

We contrasted the epigenetic profile of CD14^+^ monocytes from the deceased patient group with the one from the alive group at day 0 and at follow-up (days 5 and 15). Of the 13,398 chromatin regions tested, 959 were DAC at admission and 407 were DAC at follow-up (Fig. 4A and table S3). Two hundred twenty-two of 407 DAC regions (54.5%) at follow-up overlapped with DAC regions at day 0 (Fig. 4B and table S3). Among the deceased group, the proportion of regions with sustained chromatin changes at follow-up was higher for less accessible regions (62.2%) compared to more accessible regions (32%; Fisher exact test, *P* = 1.1 × 10^−7^; Fig. 4B). Hence, while chromatin accessibility between the two groups became less pronounced, there was still a trend to maintain accessibility status of DAC regions between admission and follow-up (Fig. 4C).

**Fig. 5.**
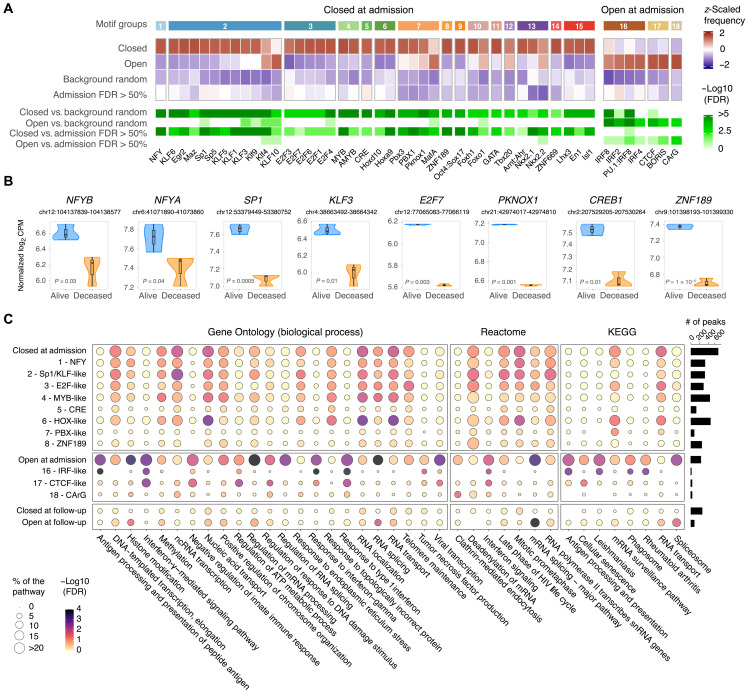
Motif enrichment, GO, and pathway analysis for DAC regions in patients with COVID-19. (**A**) Motif enrichment analysis for 959 DAC regions between deceased versus alive at admission. Of the 682 motifs tested, 46 were significantly enriched in DAC regions of deceased patients at FDR < 1% and are plotted as a heatmap. Of them, 39 motifs were more frequently observed in closed DAC and 7 more frequently in open DAC at admission. The first row indicates groups based on motif homologies (details in fig. S7). The next four rows represent the *z*-scaled motif frequency for open and closed DACs at admission followed by motif frequencies in two control groups, background random including 50 thousand genomic regions with the same length distribution as DACs and nonsignificant regions at FDR > 50% at admission. Shades of red denote higher frequency and shades of blue denote lower frequency for each motif. The last four rows show the negative log_10_ FDR for each contrast. (**B**) Suppressed promoter accessibility in deceased for eight TFs with motifs enriched in closed DAC regions at admission. Normalized log_2_ CPM for promoter peaks are plotted on the *y* axis for the deceased and alive groups. (**C**) GO and pathway enrichment analyses for DAC regions at admission and follow-up for deceased versus alive. Genes assigned to DAC regions or DACs stratified by motifs shown in (A) were used to interrogate the Kyoto Encyclopedia of Genes and Genomes (KEGG) and Reactome pathways, as well as the GO biological process term databases. Nonredundant pathways and GO terms presenting an FDR < 5% and at least five genes in one of the tested conditions are plotted, with the bubble size representing the percentage of genes in a given pathway or term and the color shades indicating the negative log_10_ FDR. The number of DACs corresponding to each peak category on the left is shown as a bar plot on the right. ncRNA, noncoding RNA.

To understand the biological mechanisms tagged by epigenetic differences in the deceased versus alive comparison, we performed DNA binding motif enrichment for DAC regions at admission. We found motifs for seven transcription factors (TFs) enriched in chromatin regions of increased accessibility in deceased patients (Fig. 5A). Among these motifs was a set of interferon regulatory factors (IRFs), known to regulate interferon responses, and CCCTC-binding factor (CTCF), which is involved in regulating transcription and chromatin architecture (Fig. 5A and fig. S7). An enrichment of motifs for 39 TFs was observed in regions less accessible in deceased patients. As many of these TFs bind to homologous motifs, we summarized the TFs in groups by similarities in their binding sites (Fig. 5A and fig. S7). The groups most represented in less accessible regions included TFs with Sp1-like and E2F-like binding motifs. These TF families not only have multiple regulatory functions including cell cycle, differentiation, and chromatin remodeling but are also associated with immune responses. Potentially as part of feedback loops, TF members of six of the top eight motif groups had their promoter region also less accessible in deceased patients (Fig. 5B and fig. S8).

Next, we performed GO and pathway analyses using genes assigned to more open or closed DAC regions at admission and follow-up. In addition, we stratified the analysis using genes assigned to DAC regions overlapping motifs for the leading TF of each group (Fig. 5C). For DAC regions more open at admission, we observed the type I interferon response pathway enriched for both IRF-like and CTCF motifs (Fig. 5C and table S4). Peaks with CTCF and IRF binding motifs were independent and assigned to different sets of genes that converged in type I interferon pathways (table S4). Other terms and pathways tagged by more accessible chromatin included mRNA splicing and ATP metabolism. The ATP metabolism pathways were mostly tagged by peaks with CTCF motifs, while spliceosome and RNA metabolic pathways were not specific to a TF motif group (Fig. 5C). Conversely, in accordance with scRNA-seq results, RNA transport and proton transporter for ATP GO terms were more frequently observed in closed chromatin of deceased patients, suggesting an unstable RNA metabolism and transport in CD14^+^ monocytes.

### Overlap of DAC with severe COVID-19 with respiratory failure genome-wide association study

**Fig. 6.**
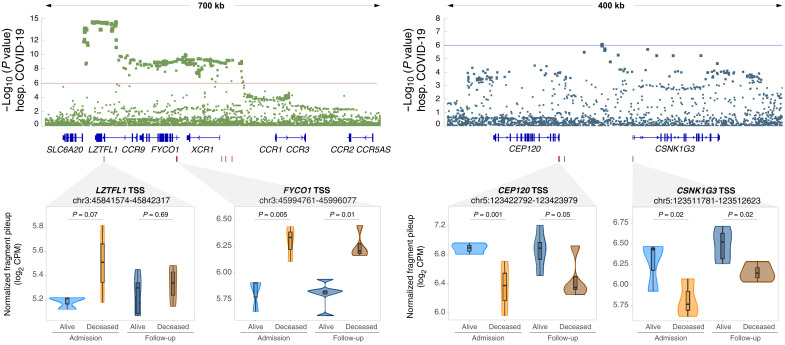
Differential open chromatin at genes located within GWAS loci for COVID-19 hospitalization. Two of the five GWAS loci for severe COVID-19 with respiratory failure contain multiple DACs in monocytes from hospitalized patients with COVID-19 (*33*). The *y* axis indicates the evidence for association of single-nucleotide polymorphisms (SNPs) (dots in the top panels) with severe COVID-19 as negative log_10_ *P* value. The *x* axis indicates the chromosomal location of SNPs with colocalizing genes shown below. A zoom-in at the four regions with DAC for the deceased versus alive contrast is shown in the lower part of the figure. Box plots with normalized quantification of pileup fragments in peaks at promoter regions for deceased and alive are shown as log_2_ CPM for patient groups at admission and follow-up.

In a previous genome-wide association study (GWAS) (*33*), two significant and three suggestive loci were associated with severe COVID-19 with respiratory failure. Of these five GWAS loci, two overlapped multiple DAC regions for the deceased versus alive at admission contrast (Fig. 6). The overlap between DAC regions and the GWAS loci was significantly not random (*P* = 0.01; fig. S9). The most significant locus associated with hospitalization in the COVID-19 GWAS encompassed the *FYCO1* and *LZTFL1* genes on chromosome 3. We observed that the promoter region of these two genes is more accessible in the deceased patient group, with the *FYCO1* promoter also maintaining increased accessibility in the follow-up (Fig. 6). For the suggestive COVID-19 protective signal observed on chromosome 5, the promoter regions of two flanking genes *CEP120* and *CSNK1G3* were less accessible in the deceased group at hospital admission and follow-up (Fig. 6).

### Differences of DNA methylation between patient groups at admission

**Fig. 7.**
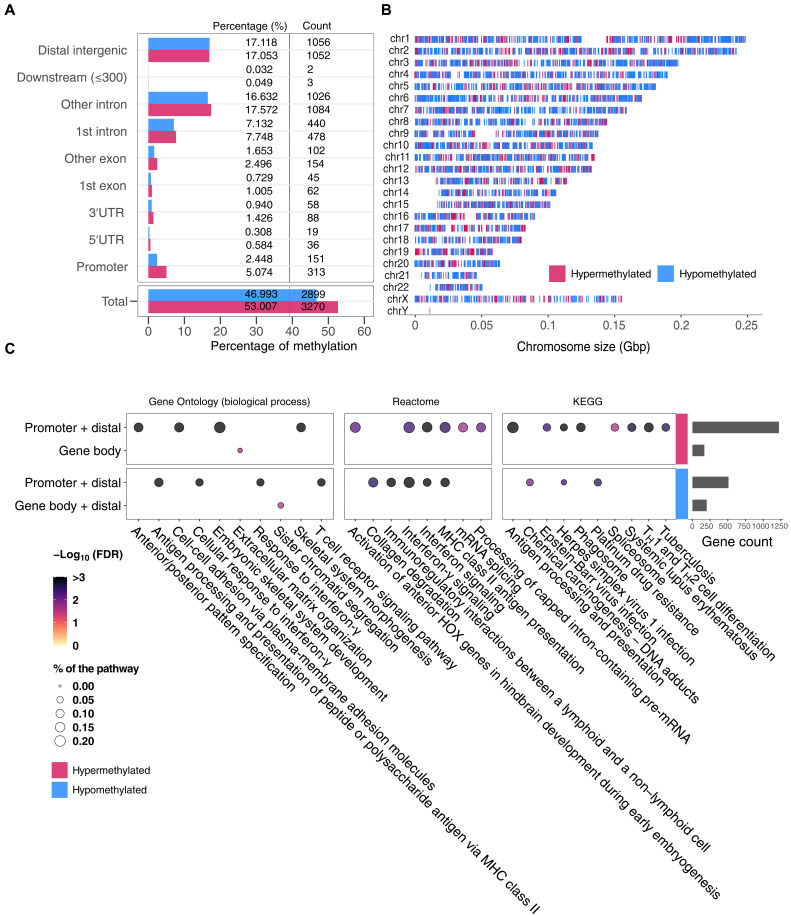
DNA methylation analysis of monocytes at admission. (**A**) Count and percentage of hypermethylated (red) and hypomethylated (blue) regions of deceased versus alive patients across annotated genomic regions. UTR, untranslated region. (**B**) Distribution of hypermethylated (red) and hypomethylated (blue) regions across chromosomes. Each vertical line indicates a DMR. (**C**) GO and pathway enrichment analyses for DMRs at admission comparing deceased versus alive patients. Genes assigned to DMRs in promoters, gene bodies, and distal regions (details in Methods) were used to search the KEGG and Reactome pathways, as well as the GO biological process term databases. Genes associated with hypermethylated DMRs are shown in the top two rows and hypomethylated DMRs in the bottom row. Nonredundant pathways and GO terms showing FDR < 5% and with at least five unique genes and DMRs in one of the test conditions are plotted; bubble size represents the FDR and color shading the percentage of genes with a corresponding significant DMR at FDR < 5%. The total number of genes per group associated with DMRs used to interrogate the databases is shown in the bar plot on the right. T_H_1, T helper 1 cell.

To further investigate changes in chromatin in CD14^+^ monocytes at admission, we performed whole-genome bisulfite sequencing (WGBS) and compared 26,893,193 CpG loci between the deceased versus alive patient groups. From the 219,626 differentially methylated loci (DMLs) with a *P* value of <0.05 (fig. S10A), we found 6169 differentially methylated regions (DMRs; fig. S10B). Approximately half (47.0%) of the DMRs were hypomethylated in deceased patients. While the majority of DMRs were located in genic and intergenic regions, 7.5% of DMRs were located in gene promoter regions (Fig. 7A). Overall, DMRs were distributed across the genome (Fig. 7B).

To evaluate the effect of these DMRs in COVID-19 pathogenesis, we performed a TF binding motif enrichment analysis as implemented for DAC regions and found no significant enrichment at FDR < 0.01. Next, we performed GO term and pathway analyses on the basis of genes tagged by promoter, distal, and gene body DMRs. Using hypermethylated DMRs, located at promoters or tagging distal genes, we observed spliceosome-related pathways and antigen processing and presentation terms significantly enriched in deceased patients (Fig. 7C and table S5). In addition, for DMRs in gene body, several pathways including the extracellular matrix (ECM) organization were significant. Moreover, GO terms and pathways including sister chromatid segregation were enriched in gene body and/or distal DMRs. No enrichment was found using hypomethylated DMRs located in the gene body and hypermethylated DMRs located in the gene body combined with distal DMRs.

A recent study showed that changes in gene expression in response to infections occur before detectable alterations in the methylome (*34*). Therefore, we hypothesized that methylation changes might increase only at advanced disease stages after the admission. Using the same deceased versus alive design applied to follow-up samples, we detected 486,512 DMLs (*P* < 0.05) (fig. S10C). DMLs were then used to identify a total of 21,298 DMRs (fig. S10D). The intersection between the DMRs found at admission and follow-up suggested that approximately 40% of DMRs were conserved during disease progression (fig. S11A). When we conducted GO and pathway analyses for the genes tagged by DMRs at follow-up, we observed 19 (24.1% from admission) pathways and GO terms that were already enriched at admission and thus maintained during disease progression, including spliceosome-related pathways and positive regulation of telomere maintenance (fig. S11B and table S6). In addition, several pathways and GO terms including chromatin organization, RNA localization, and modifications were observed exclusively at follow-up. Although we did not observe notable methylation changes at admission, we showed a significant increase of GO terms/pathways tagged by methylation state during follow-up that were aligned with those tagged by transcriptomic and chromatin changes. This suggested that differences in DNA methylation became more pronounced with increasing time of COVID-19 hospitalization and that these changes might be a downstream consequence of the host response to SARS-CoV-2.

### Data integration and identification of candidate drugs

We performed an ingenuity pathway analysis (IPA) including all DEGs for the deceased/alive contrast at admission. This analysis resulted in more than 1500 candidate drugs. To narrow down the list of candidates, we focused the IPA on 62 up-regulated DEGs with a corresponding increased DAC peak in deceased patients at admission. The IPA identified 53 candidate drugs/compounds to treat severely ill patients with COVID-19 (fig. S12). The candidate drugs identified are mainly used to treat cancers and/or inflammatory conditions. Fifty of these drugs could be divided in 16 classes, while 3 presented multiple biological mechanisms (fig. S12). Among the 53 candidates detected were dexamethasone and baricitinib, drugs currently used for the treatment of hospitalized severely ill patients with COVID-19 (fig. S12).

**Fig. 8.**
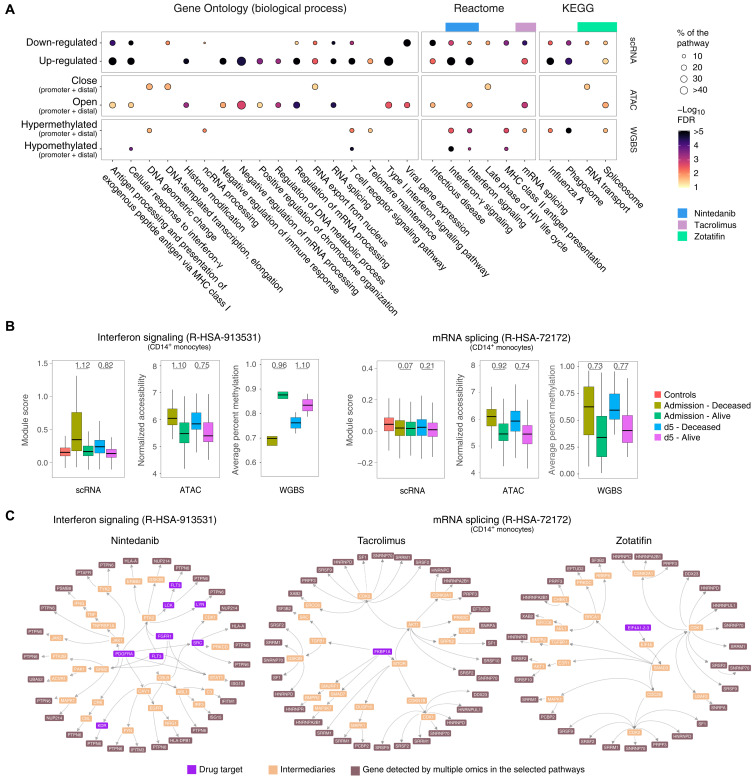
Candidate drugs to treat hospitalized patients with COVID-19. (**A**) Pathways and GO terms identified by differential transcriptomic, chromatin accessibility, and/or DNA methylation are shown in the bubble plot. The bubbles indicate pathways or terms with more than 10 genes and FDR < 5%. The bubble shades represent the −log10 (FDR), and the size indicates the proportion of genes in the pathway or term that were significantly different between deceased and alive. Tacrolimus, zotatifin, and nintedanib were identified as candidate drugs via interaction with pathways significant in all three omics. (**B**) Evolution of transcriptome and epigenetic changes in the interferon signaling and mRNA splicing pathways. DEGs were used to generate a module score representing the overall expression level of genes per pathway in CD14^+^ monocytes. The normalized average log_2_ CPM of DAC represents the pathway overall accessibility profile. The average percentage of DNA methylation for DMRs indicates the DNA methylation status of combined promoter and distal regions. The quantifications shown were derived at admission and the day 5 time points for the deceased and alive contrasts. Significance of differences between deceased and alive at different time points was tested using a Wilcoxon rank test and Cohen’s *d* effect size estimation as shown at the top for every significant variation at *q* value < 0.05. (**C**) Protein-protein interaction network linking the biological target of candidate drugs with proteins encoded by genes in the pathways identified by multiple omics. The proteins targeted by the candidate drugs were accessed in DrugBank and intersected by means of the OmnipathR protein-protein interaction database. Proteins targeted by the drugs zotatifin, tacrolimus, and nintedanib are shown in purple. Genes tagged by at least two assays in the interferon signaling (nintedanib) and mRNA splicing (tacrolimus and zotatifin) pathways are shown in brown. Intermediate genes marked in orange show the cascade from drug target to proteins encoded by genes in the tagged pathways.

We observed a high level of consistency for GO/pathway terms between chromatin accessibility and gene expression. Specifically, in the deceased patient group, most of the terms tagged by up-regulated genes were likewise detected by increased chromatin accessibility (Fig. 8A). The concordance of epigenetic and transcriptomic changes was often observed for individual genes. For example, genes such as *IFITM2*, *IFITM3*, *IFI6*, and *ISG15* included in the “type I interferon signaling” term were up-regulated in deceased patients at admission and also displayed increased promoter accessibility (fig. S13). As the disease advanced, both transcriptional and chromatin structure differences in the deceased versus alive contrast attenuated (tables S1 and S3). Significant differences in splicing-related pathways between deceased versus alive patients were observed at the gene expression, chromatin accessibility, and DNA methylation levels (Fig. 8A). Moreover, RNA splicing–related terms were among the most significant pathways detected by open DACs and hypermethylated DMRs at follow-up, suggesting that deceased patients could not suppress splicing-related mechanisms (Figs. 5C and 6C). The observation that reduced chromatin accessibility of mRNA splicing–related genes improved survival was also highlighted by corresponding transcriptomic changes (Fig. 8A).

The identification by different assays of the same differentially activated pathways in cells from COVID-19 survivors and deceased implicates these pathways as determinants of prognosis and thus supports the validity of these pathways as targets for pharmacological intervention. In an additional effort to support the results of the data integration, we also searched for already published datasets, consistent with our own study. We were unable to identify datasets that could be used for replication of monocyte chromatin structure and DNA methylation. We identified two datasets of scRNA-seq that considered patient phenotypes compatible with our enrollment criteria (*35*, *36*), i.e., inclusion of severely ill patients with COVID-19, reporting of OS scores, and disease outcomes. Unfortunately, the datasets still differed in the technology used and the time of enrollment after diagnosis/onset of symptoms, reducing comparability of the results (fig. S14A). Nevertheless, we observed similar transcriptional changes when comparing severely ill patients and healthy controls. Moreover, “mRNA splicing” was up-regulated in CD14^+^ monocytes of deceased patients from the study of Wilk *et al.* (*35*), while the “response to interferon” pathway was up-regulated in CD14^+^ monocytes of “deceased” patients in the study of Schulte-Screpping *et al.* (*36*) (fig. S14B).

To directly compare the transcriptomic and epigenetic results for the interferon signaling and mRNA splicing pathways, we summarized the scRNA module score using all CD14-expressing monocytes (resting, activated, and intermediate; Fig. 8B) to create a pool of cells that is equivalent to the CD14 monocytes enriched by our magnetic isolation method. We calculated the average log_2_ count per million (CPM) normalized accessibility of DAC regions (ATAC) and the average percent of methylation for DMRs (WGBS) assigned to genes in the selected pathways both at day 0 and at day 5 (Fig. 8B). For the interferon signaling pathway, we observed a multilayered impact. Genes assigned to the interferon pathway had higher expression, accessibility, and more hypomethylated regions for deceased patients at admission (Fig. 8B). For the mRNA splicing pathway, we observed stronger epigenetic than transcriptomic effects in CD14^+^ monocytes (Fig. 8B). However, stronger transcriptomic effects for the mRNA splicing pathway were observed in T and B cells (fig. S15A). We observed more hypermethylated DMRs associated with deceased patients at day 0. DNA hypermethylation in promoter regions is associated with repression of gene expression, while distal DNA methylation can have different regulatory effects. For the mRNA splicing pathway genes, 93% of the hypermethylated DMRs were distal, while for the interferon signaling pathway, 60% of the DMRs were in the targeted gene promoter. We searched for existing drugs interacting with pathways and GO terms detected by at least two experimental approaches (details in Methods). We found that tacrolimus, a calcineurin inhibitor via the immunophilin FKBP1A (FK506 binding protein prolyl isomerase 1A) with properties similar to cyclosporine, interacted with multiple genes and hubs in the Reactome mRNA splicing pathway (fig. S15A). By assessing the Kyoto Encyclopedia of Genes and Genomes (KEGG) drug interactome, we identified zotatifin, a selective eIF4A (eukaryotic initiation factor-4A) inhibitor, as an additional repurposed candidate drug targeting the spliceosome. When evaluating the Reactome interferon signaling pathway, we detected nintedanib, a triple angiokinase inhibitor used in the treatment of pulmonary fibrosis, as a candidate drug interacting with multiple genes and hubs of these pathways (*37*). To further assess the interaction network between the candidate drugs and their known biological targets, we intersected OmniPath’s protein-protein interaction database with the drugs’ known biological targets according to DrugBank (Fig. 8C; details in Methods). Using proteins encoded by genes detected by at least two omics assays as “proteins of interest,” we constructed the protein-protein interaction network for tacrolimus and zotatifin starting at their respective drug target and ending at proteins of interest in the mRNA splicing pathway. The same strategy was applied for the multiple targets of nintedanib and proteins of interest of the interferon signaling pathway. Because the deceased patients exhibited an overactivation of these pathways, drugs interfering with the excessively activated pathways provide promising candidates for further study.

## DISCUSSION

We applied a systems biology approach to study peripheral immune cells of severely ill patients with COVID-19 at hospital admission and identified monocytes as key effector cells of COVID-19 innate immunity. The focus of our study was the identification of host response pathways that differed significantly between patients who would recover from COVID-19 and those who would succumb to the disease. To identify host response pathways that lead to recovery or death, we used single-cell transcriptomics and epigenomics approaches. The importance of epigenetic changes of host cells for the viral life cycle had previously been suggested by the detection of histone and chromatin modification during SARS-CoV-2 infection (*38*). We identified response to interferon and RNA metabolism/splicing pathways as significantly overactivated in patients with poor outcomes. These pathways constitute promising tractable targets for pharmacological intervention to reduce mortality of hospitalized patients with COVID-19. The hallmark of the life-threatening COVID-19 is severe pneumonia resulting in structural damage to lung alveoli, emphasizing the role of pulmonary immune responses. However, scRNA-seq of bronchoalveolar lavage cells from patients with COVID-19 identified an excess of inflammatory monocytes in multiple studies. Infiltrating monocytes can become part of a self-enforcing inflammatory loop involving tissue-resident alveolar macrophages and T cells (*39*). It is plausible that monocytes and T cells with overactivated interferon response and RNA metabolism pathways will exaggerate this loop, resulting in inflammatory tissue damage that will lead to the recruitment of profibrotic monocytes and poor outcomes (*40*).

Our study design was distinct from previous studies comparing patients with severe COVID-19 to controls or mild patients (*41*–*45*) or studies comparing COVID-19–infected patients to other viral diseases (*45*–*47*). With our design, we were able to confirm well-recognized COVID-19 severity characteristics such as type I interferon dysregulation (*48*–*50*) or the systemic up-regulation of S100A8/A9 genes in monocytes (*42*). Furthermore, our results showed a strong correlation of PBMC composition with disease progression. Critically ill patients with poor prognosis showed a significant reduction of T cells and a significant increase of monocytes, consistent with previously reported findings in patients suffering from severe COVID-19 (*43*, *51*, *52*). The changes in the proportion of T cells and monocytes that we observed were consistent with a declining role of innate immunity (monocytes) and a more prominent role of T cell immunity at the advanced stage of COVID-19 disease. In monocytes and T cells, we detected a transcriptional signature at hospital admission, which strongly correlated with disease evolution. At hospital admission, naive CD4^+^ T cells and T_cm_ cells of patients with poor prognosis were strong expression of genes enriched in apoptosis, response to oxygen levels, proteostasis, and ER stress response terms, which are associated with early stages of cell death (*53*, *54*). In monocytes of these patients, we observed a signature of reduced expression of genes involved in mitochondrial ATP synthase (mainly genes of the complex V), MHC, and ribosome and translation initiation terms. Mitochondrial ATP activity is associated with cell cycle regulation, and a decrease of ATP level is associated with cell proliferation (*55*). Overall, the concordance between the transcriptional signatures at an earlier stage of the disease and the changes of PBMC composition as the disease evolved reinforced the importance of early care treatment decisions to increase COVID-19 patient survival.

The extent of transcriptional variations detected between deceased and alive patients with COVID-19 at hospital admission, and the continued transcriptional dysregulation with worsening disease at follow-up, supports the pivotal role of monocytes in COVID-19 severity and disease prognosis, as seen by others (*56*). The key role of monocytes for COVID-19 pathogenesis was also demonstrated by our finding that two of the five GWAS loci for respiratory failure colocalized with chromatin having increased accessibility in monocytes from patients who succumbed to the disease (*33*).

The most significant GWAS hit for COVID-19 severity localizes to chromosome 3 in a region of high linkage disequilibrium. Among the genes found in the GWAS locus, *LZTFL1* is a strong functional candidate gene. *LZTFL1* encodes a cytosolic leucine zipper protein involved in cellular trafficking, regulation of ciliogenesis, and ciliary cell function (*57*, *58*). The COVID-19 GWAS risk allele on chromosome 3p21.31 (rs17713054) creates a CEBPB (CCAAT enhancer binding protein beta) motif resulting in an enhancer that directly interacts with the *LZTFL1* (leucine zipper transcription factor like 1) promoter. LZTFL1 is a regulator of epithelial-mesenchymal transition (EMT), which is part of the host pulmonary anti–SARS-CoV-2 response. Increased expression of *LZTFL1* inhibits EMT and therefore blunts antiviral host responses (*58*). Postmortem lung biopsies of patients with COVID-19 showed widespread epithelial disorganization with EMT signatures, which correlated with a rapid progression from symptom to death (*58*). The higher promoter accessibility for *LZTFL1* in CD14^+^ monocytes of deceased patients at admission is in agreement with the observation that increased *LZTFL1* expression is detrimental in COVID-19 pathogenesis. While increased pulmonary influx of profibrotic monocytes in advanced COVID-19 is a major contributing factor to poor disease outcomes (*40*), the role of *LZTFL1* in monocyte physiology is unknown. However, the critical role of infiltrating inflammatory monocytes in COVID-19 pathology does provide a strong rationale for the study of circulating monocytes in patients with severe COVID-19. Given that *LZTFL1* may not be the only gene involved in COVID-19 vulnerability, our data implicating *FYCO1* as an additional monocyte candidate gene of the chromosome 3 GWAS susceptibility locus also deserve further consideration. For example, three coding variants of *FYCO1* are associated with COVID-19 severity (*59*). FYCO1 (FYVE and coiled-coil domain autophagy adaptor 1) is a Rab7 adapter protein implicated in vesicle transport during autophagy, a well-established host defense mechanism in host response to viruses (*60*).

We also showed that monocyte DNA methylation changes linked with disease severity are associated with genes in the ECM and proteolysis pathways. The implication of the ECM in disease severity could highlight the damage to the structure and function of SARS-CoV-2–infected tissues (*61*), and the alteration of proteolysis pathways is consistent with the role of host proteolytic enzymes in viral replication and assembly (*62*). Notably, by combining monocyte transcriptomics with chromatin accessibility and DNA methylation, we identified among patients with equivalent clinical severity at admission the activated pathways that tagged those destined to succumb to COVID-19. Most prominent were pathways related to RNA splicing, metabolism and transport, chromatin architecture, and cellular response to interferon. While the pathways were identified by a multiomics approach in CD14^+^ monocytes, the corresponding transcriptomic changes could be tracked to B, T, and NK cells, indicating that their contribution is not limited to monocytes.

Motifs of the NFY family were the most significantly enriched TFs in closed regions of deceased patients at admission. NFY is a heterotrimeric complex composed of the NF-YA, NF-YB, and NF-YC subunits (*63*). The NFY complex has nucleosome-like properties and regulates chromatin accessibility at transcription starting site (TSS) containing CCAAT sequences because of its similarity with the H2A/H2B nucleosome or via regulation of histone acetyltransferases (*63*–*65*). The NFY complex maintains regions upstream of TSS in a nucleosome-depleted state (i.e., open) while simultaneously protecting this accessible region from aberrant transcription initiations (*65*). A loss of NFY binding decreases promoter accessibility through nucleosomal encroachment. We observed an enrichment of NFY motifs in closed chromatin, which could indicate a lack of NFY TF binding and suppression of pathways targeted by these chromosomal regions. Another consequence of the lack of NFY binding is a relocation of the transcription preinitiation complex resulting in the generation of aberrant extended transcripts (*65*). The aberrant transcripts undergo translation and splicing, potentially disrupting RNA metabolism, a mechanism that was altered in deceased patients with COVID-19. In our data, we observed the “DNA-templated transcription” and “RNA transport” pathways enriched for closed chromatin with NFY motifs and a down-regulation of RNA expression in these pathways (Fig. 8A), which supported the concept that the NFY complex was an important regulator of chromatin structure and RNA metabolism. Notably, in a coordinated effect, the promoters of the NFYA and NFYB genes were less accessible in deceased patients. Hence, the loss of NFY promoter accessibility might result in depletion of the NFY complex, which would be expected to have a strong impact on the genomic landscape and transcriptomic host response against SARS-CoV-2. Binding motifs for the TF specificity protein 1 (SP1) were the second most enriched motif in the closed chromatin of deceased patients. NFY and SP1 regulate the expression of genes encoding heat shock proteins (HSPs) in response to cellular stress (*66*). In line with this TF function, closed chromatin with SP1 motifs was enriched for genes encompassed in the GO terms for “response to endoplasmic reticulum stress” and “response to topological incorrect protein” (Fig. 5C). This finding suggested that deceased patients had difficulties coping with the stress that CD14^+^ cells were undergoing due to a defect of HSPs homeostasis leading to an accumulation of misfolded proteins.

The functional pathways identified by the combined transcriptomics and epigenetics approach in our study had been previously implicated in COVID-9 pathogenesis. However, our study design pinpointed the differential activity of the pathways at the junction when potentially life-saving clinical treatment decisions for critically ill patients need to be made: when they are admitted to the intensive care unit. The involvement of interferon pathways in the COVID-19 pathogenesis has been well established (*48*–*50*). However, while we observed an increased interferon response in deceased patients, we cannot ascribe these abnormal responses as causal to the prognosis; alternatively, they may reflect a host compensatory mechanism (e.g., to uncontrolled viral replication). In contrast, RNA splicing by host cells is a key step of viral replication. SARS-CoV-2 encompasses 16 nonstructural proteins (NSP1 to NSP16) that are required for the virus to hijack the host transcriptional machinery. Viral NSP16 binds to the U1 and U2 spliceosome complex to negatively regulate host mRNA maturation (*67*). In addition, in vitro challenge of Vero E6 cells with SARS-CoV-2 showed increased phosphorylation of proteins involved in RNA splicing–related pathways, positioning splicing as a critical mechanism of COVID-19 pathogenesis (*38*). The increased splicing activity observed for deceased patients might benefit viral replication and impair host immune responses. For instance, splicing disruption was shown to impair interferon responses to SARS-CoV-2 via intron retention (*67*). We found that genes encoding proteins involved in RNA transport and signal recognition particle (SRP)–dependent protein targeting to the membrane were down-regulated and less accessible in patients that would succumb to COVID-19. This may reflect the binding of viral NSP8 and NSP9 to the 7SL RNA component of the SRP, which interferes with protein trafficking (*67*).

RNA splicing typically occurs in the nucleus while SARS-CoV-2 replicates in cytoplasmic organelles (*68*). However, that does not limit the importance of the host splicing machinery for viruses that replicate in the cytosol. Infection with SARS-CoV-2 has a marked impact on spliceosome functions in different cell types (*14*, *67*, *69*). Viruses can disrupt host mRNA splicing by triggering the nucleocytoplasmic translocation and sequestering of spliceosome components. In Caco-2 cells, 14 of 25 spliceosome components that were increased after infection with SARS-CoV-2 directly bound to SARS-CoV-2 proteins. In these cells, inhibiting splicing with pladienolide B prevented viral replication at concentrations nontoxic to humans (*14*). In addition, the SARS-CoV-2 NSP16 protein inhibited mRNA splicing in human embryonic kidney (HEK) 293T cells, leading to a diminished interferon response via intron retention (*67*). In severely ill patients with COVID-19, we observed an excessive interferon response; therefore, targeting the splicing machinery could help balance the immune system in these critical patients. In SARS-CoV-2–infected NHBE (normal human bronchial epithelial) cells, an increase in alternative splicing was observed for genes encoding ribosomal proteins, suggesting that the virus rewired the posttranscriptional mRNA network (*69*). For example, the SARS-CoV-2 NSP1, critical for viral replication by suppressing host gene expression via blockage of the ribosomal mRNA channel (*70*), bound several pre-mRNA splicing factors (*71*). An additional noteworthy interaction of NSP1 was with eIF4G. eIF4G forms a complex with eIF4A (*72*), which is the specific target of zotatifin, one of the drugs described in our study. SARS-CoV-2 also affects splicing with its circular RNAs. SARS-CoV-2 circular RNAs down-regulated genes associated with metabolic processes and up-regulated genes associated with cellular responses to oxidative stress and centrosome localization, thus linking infection with changes in the cell nucleus (*73*). Together, these observations support the importance of SARS-CoV-2 interference with host mRNA splicing in COVID-19 either via viral proteins or viral circular RNAs. While long-term therapies targeting mRNA splicing might result in adverse effects, inhibition of mRNA splicing in the acute phase of COVID-19 hospitalization might help to reduce viral replication and favor life-saving host immune responses.

Our work demonstrates the strengths of integrative epigenomics analyses to characterize the biological effectors that influence the survival of severely ill patients with COVID-19 and the potential drug targets associated with them. Nevertheless, there are limitations to our study. The actual samples used in this study have been collected from patients at the early stage of the SARS-CoV-2 pandemic. Since then, the standard of care for severely ill patients with COVID-19 has drastically evolved, and we cannot know whether this would affect our results. An exhaustive search for datasets that conform to the criteria of the present study identified only two studies by Wilk *et al.* (*35*) and Schrepping-Schulte *et al.* (*36*) that could be used for a comparative analysis. Unfortunately, the use of different technologies prevented the integration of all datasets for a comparative analysis, and we conducted per-study comparisons. Besides the effect of different technologies, we think that the time from symptoms onset/diagnosis to sampling that was shortest for Wilk *et al.* (*35*) and longest for Schulte-Schrepping *et al.* (*36*) had the largest impact on the comparative analysis. An effective interferon response early during SARS-CoV-2 infection supports a positive outcome, whereas sustained interferon responses are associated with worsening disease. This suggests, in line with our observations, a detrimental role for strong interferon responses only at more advanced disease stages. Combined, the two published datasets selected for replication and our own study highlight the dynamic transcriptional changes at play during the course of SARS-CoV-2 infection and emphasize the critical role of sampling time. On the basis of these considerations, we propose the day of hospitalization (when patients reach an OS of 7) as a critical and privileged window for therapeutic intervention using the drugs identified by our study.

Our study design further enabled the identification of candidate drugs to reduce mortality among critically ill patients with COVID-19. Using drug-protein and protein-protein interaction databases, we identified three drugs (tacrolimus, zotatifin, and nintedanib) that target pathways that are differentially activated at hospital admission of critically ill patients and that discriminate between eventual survivors and deceased. Tacrolimus and zotatifin may exert a dual role by interacting with the spliceosome and with NSPs from SARS-CoV-2 (*16*, *74*). Although tacrolimus is known as a calcineurin inhibitor, it also interacts with U2 RNA components of the spliceosome and was shown to bind to NSP1, a protein that SARS-CoV-2 uses to disrupt protein translation in infected cells (*74*). Additional support for tacrolimus as a candidate drug are the observation that cyclosporin (a molecule with similar properties to tacrolimus) reduced mortality in hospitalized patients and the increased survival from COVID-19 in liver transplant recipients treated with tacrolimus (*11*, *75*, *76*). Moreover, tacrolimus regulates immune gene–priming long noncoding RNAs (lncRNAs). For example, monocytes pretreated with tacrolimus do not trigger UMLILO expression when stimulated with β-glucan (*77*). UMLILO is an immune gene–priming lncRNA that plays a key role in the activation of trained innate immune responses. Decreasing UMLILO-dependent immune activation with either tacrolimus or other NFAT inhibitors such as cyclosporin A may contribute to control the excessive inflammatory response in severe COVID-19. Zotatifin, a member of the class of plant-derived cyclopenta[b]benzofurans compounds named rocaglates, inhibits specifically the RNA helicase eIF4A that is part of the EJC/TREX (exon junction and mRNA transport) complex of the spliceosome. Moreover, zotatifin is part of a network encompassing NSP13 and interacts with NSP9 (*16*, *78*). The mechanism of action proposed for zotatifin is via the reduction of viral infectivity and viral protein biogenesis, and it is under clinical trial to treat COVID-19 (NCT04632381). Similarly to zotatifin, the rocaglate CR-1-31-B was shown to potently inhibit SARS-CoV-2 replication at low nanomolar doses in vitro and ex vivo (*79*). Both tacrolimus and zotatifin were among the drugs identified by in vitro and in silico models as candidate drugs to treat COVID-19 (*16*, *17*, *74*). Nintedanib is a drug used to treat pulmonary fibrosis. In a small clinical trial, nintedanib did not show significant improvement in the survival of severe pneumonia induced by COVID-19 (*80*). However, nintedanib shortened the mechanical ventilation time compared to the placebo group (*80*). Given that severely ill patients with COVID-19 may require prolonged respiratory support, nintedanib could be considered as an adjunct therapy with other drugs. In our study, we confirmed the potential of tacrolimus, zotatifin, and nintedanib to treat critically ill patients with COVID-19 at hospital admission, providing required preclinical data to support the testing of these drugs in controlled clinical studies.

## METHODS

### Patients

Participants enrolled in the present study were individuals quantitative polymerase chain reaction (PCR)–diagnosed with SARS-CoV-2 who were admitted to the MUHC (Montreal, Quebec, Canada) and recruited to the Canadian Treatment for COVID-19 (CATCO) clinical trial (https://clinicaltrials.gov/ct2/show/NCT04330690) between March and April 2020. CATCO is a randomized, open-label, controlled trial and represents the Canadian arm of the WHO SOLIDARITY trial. Blood samples were collected under MUHC Research Ethics Board protocol 10-256. Patient clinical progression was evaluated using the WHO Clinical Progression Scale, which represents a minimal common outcome measure set for COVID-19 clinical research (*32*). Briefly, it provides a measure of illness severity from not infected (ordinal score of 0) to deceased (ordinal score of 10) (*32*). All patients received standard of care except one patient who received lopinavir/ritonavir (Kaletra). During the conduct of this study, the standard of care at our institution consisted solely of supplemental oxygen as required, antithrombotic prophylaxis, and supportive care in the intensive care unit when needed. On the basis of the outcome of hospitalization, patients were classified as deceased if they died during hospitalization and alive if they were discharged and considered recovered. At admission, all patients were severely ill (OS of 7). To follow disease evolution, disease severity during hospitalization was classified in three categories according to the WHO ordinal score: moderate (OS of 4 or 5), severe (OS of 6 or 7), and critical (OS of 8 or 9). Blood samples were collected through standard venipuncture in standard EDTA blood collection tubes, and PBMCs were obtained through Ficoll density centrifugation with SepMate tubes (STEMCELL Technologies) and kept frozen in 10% dimethyl sulfoxide and 90% fetal bovine serum until analysis. The study was approved by the MUHC Research Ethics Board protocol 10-256.

### scRNA library preparation

Single-cell PBMC suspensions were loaded on the Next GEM Chip G (PN-1000120) together with the Next GEM Single Cell 3′ GEM Kit v3.1 (PN-1000121), and single cells were captured on a 10x Genomics Chromium controller. Complementary DNAs (cDNAs) were generated following the 10x Genomics protocol. cDNAs were size-selected using SPRIselect beads from Beckman Coulter (B23318), and their quality was checked with the Bioanalyzer High Sensitivity DNA Kit from Agilent (5067-4626). One-quarter of the total cDNA was used to generate libraries using the Chromium Next GEM Single Cell Library Kit (PN-1000121) and barcoded using the Single Index Kit Set A (PN-111213) following the 10x Genomics protocol. Libraries were size-selected using SPRIselect beads, and their quality was checked with the Bioanalyzer High Sensitivity DNA Kit. Libraries size was centered at 450 base pairs (bp) and paired-end sequenced on NovaSeq 6000 S4 chips.

### ATAC-seq and WGBS library preparation from CD14^+^ monocytes

CD14^+^ monocytes were isolated from PBMCs with magnetic beads using the STEMCELL EasySep Human CD14 Positive Selection Kit II (17858). Viability was assessed with trypan blue staining, and only samples with a viability of >85% were used. We then assessed chromatin accessibility using the ATAC-seq method using 50,000 CD14^+^ monocytes (*81*). Briefly, CD14^+^ monocytes were permeabilized with lysis buffer containing 0.05% IGEPAL, and subsequently obtained nuclei were incubated with Tn5 transposase from Illumina (20034198) for 30 min at 37°C. Transposed DNAs were purified using the QIAGEN MinElute PCR Purification Kit, and the ATAC-seq libraries were amplified for a total of 8 to 12 PCR cycles using the NEBNext High-Fidelity 2X PCR Master Mix (M0541) and the Nextera XT Index Kit v2 from Illumina (FC-131-2001 and FC-131-2004). Final libraries were purified using the sparQ PureMag Beads from QuantaBio (95196-060), and their quality was checked with the High Sensitivity DNA Kit from Agilent (5067-4626). Libraries’ fragment sizes were mostly distributed between 200 and 1000 bp. Last, libraries were 150 bp paired-end sequenced on a NovaSeq 6000 S4 chip.

Genomic DNA was extracted from CD14^+^ monocytes using a QIAGEN blood and tissue kit (69504). WGBS libraries were prepared using the NxSeq AmpFREE Low DNA Library Kit. In summary, genomic DNA was sheared using a Covaris E220 instrument. After shearing, cleanup was carried out using AMPure XP beads (0.8×) followed by end repair and A-tailing to allow for adaptor ligation. After ligation, cleanup was made using AMPure XP beads (1×) before proceeding to the size selection (AMPure 0.75×). Another bead cleanup (1× AMPure) is realized to remove primer dimers. Bisulfite conversion was made using the EZ-96 DNA Methylation-Gold MagPrep from Zymo Research. The bisulfite-converted sample libraries were amplified using litigation-mediated (LM)–PCR followed by a postamplification cleanup (AMPure 1×). Fifteen WGBS libraries were 150 bp paired-end sequenced on a NovaSeq 6000 S4 chip.

### scRNA-seq clustering analysis

scRNA-seq sequence reads quality was assessed with BVAtools (https://bitbucket.org/mugqic/bvatools/src/master/). Cell Ranger v3.0.1 was used for mapping the reads to the hg38 human reference genome assembly, filtering, and counting barcodes and unique molecular identifiers (UMIs), resulting in a list of UMI counts for every gene in every cell. To increase the robustness of our data analysis, we also integrated in the analysis four external healthy donor PBMC controls (three males and one female) available from 10x Genomics (three PBMC CITE-seq (cellular indexing of transcriptomes and epitopes by sequencing) and one PBMC scRNA-seq). Data from two other healthy male donors (34 and 65 years old) were generated on-site, where also included (*82*). On the basis of the barcode and UMI counts, we extracted the summary statistics for the number of genes expressed per cell and for the number of UMIs per cell and applied our cell quality and doublet filtering pipeline (*83*). Cells with >20% mitochondrial genes were excluded. Low-quality cells and doublets were filtered out by excluding cells falling outside the [−1 SD to +2.5 SD] interval for UMI and gene count distributions. Events coexpressing either of the cell-specific markers TRBC1 (T cell receptor beta constant 1), LILRA4 (leukocyte immunoglobulin-like receptor subfamily A member 4), CD14, and CD79A were considered as doublets and excluded. The DoubletFinder package was used to identify and filter out the remaining cell doublets (*84*). Once dead cells and doublets had been removed, the patients with COVID-19 and control samples were analyzed jointly with the Seurat v4 R package, and cell clustering was performed by the UMAP dimension reduction method using the first 25 principal components with the most variable genes but excluding mitochondrial and ribosomal protein genes (*85*, *86*). This analysis identified four major populations divided into 22 distinct subpopulations, based on the marker genes for each cluster determined by MAST (model-based analysis of single-cell transcriptomics) and validated by TotalSeqB surface protein tagging by CITE-seq controls (*87*).

### scRNA-seq cellular composition analysis

To estimate the cellular composition, the PBMC lineage proportions were estimated for the four major populations and the 22 subpopulations. The number of cells composing each lineage inside a particular sample was normalized by the overall number of cells in that sample. The distribution of the sample-specific lineage proportion was contrasted overall between patients and healthy controls, at admission between patients retrospectively classified as deceased and alive, and at later time points between patients operationally defined as “moderately” and “critically” ill. Pairwise *t* test comparisons for each contrast in each lineage were used to estimate the impact of COVID-19 on the cellular composition. FDR-adjusted *P* values (e.g., Storey and Tibshirani *q* value) were used to assess the significance of these comparisons.

### scRNA-seq DGE analysis

Gene expression changes across the two comparisons deceased versus alive at day 0 and critically versus moderately ill at follow-up (days 5 and 15) were evaluated in each lineage using the MAST approach adjusting for age and sex covariates (*87*). Adjusted *P* values were estimated using the Benjamini-Hochberg (BH) procedure. Every significant DEG (adjusted *P* value ≤ 0.05) was extracted as down- or up-regulated on the basis of log_2_FC value and used for downstream analysis. All differential expression results are provided in table S1.

### scRNA-seq pathway enrichment analysis

Enrichment analyses for GO biological processes were performed separately on up- and down-regulated DGE genes and for each cell subpopulation using the enrichGO function of the clusterProfiler R package v3.13 (*88*). Enriched results were filtered to keep only GO terms for biological processes at level 5. Hierarchical level classification was obtained on the basis of the directed acyclic graph defined by the GO Consortium provided through the GO.db R Package version v3.13. In addition, only enrichment results showing a BH adjusted *P* value of ≤1 × 10^−5^ supported by at least five DEGs were considered. Enrichment heatmaps were generated on the basis of these selected results using the pheatmap R package v1.0.12. Lineage enrichment clustering was generated using the complete-linkage clustering method integrated in the pheatmap function. All level 5 enrichment results are provided in table S2 (*89*).

### scRNA-seq module score analysis

Genes contributing to the enrichment of a GO category were extracted to form a transcriptional active module corresponding to that category. Module scores were estimated with the Seurat’s AddModuleScore function, using default settings (*90*). The module score is determined for each cell by computing the mean transcriptional activity of the genes composing the module minus a mean expression extracted from a set of control genes. Control genes were randomly sampled from bins defined using the observed level of expression for the genes in the module. Module gene composition is available in table S7.

### ATAC-seq data analyses

The 15 ATAC-seq libraries were checked for read quality, and low-quality reads were removed and adaptor sequences trimmed. Reads were then aligned to the human genome (hg38) using Burrows Wheeler aligner (BWA) default parameters (*91*). PICARD v2.18.9 was used to mark duplicates and assess the fragment length distribution (https://broadinstitute.github.io/picard/). Next, samtools v1.9 was used to remove reads aligned to mitochondria or alternative contigs (*92*). AlignmentSieve from DeepTools was used to filter for unique paired reads (with --samFlagExclude 1804), to remove ENCODE hg38 blacklisted regions, and to select fragments between 40 and 2000 bp in length (*93*, *94*). The average fragment size per library was 219 bp (SD of 16.3 bp) with a mean depth of 89.3 (SD of 32.8) million fragments. All libraries presented a periodic nucleosome pattern and passed quality control. Chromatin accessibility was determined using MACS2 v2.2.6 callpeak with *q* value < 0.05 in BAMPE mode (*95*). We merged overlapping narrow peaks detected in at least two samples with bedtools v2.26 (*96*). The intersected peak set consisted of 13,892 genomic regions with an average width of 978 bp. These peaks were selected for the count-based quantification of accessible chromatin. For each library, featureCounts v1.6.3 was used to count the number of unique fragments overlapping the targeted regions and determine the FRIP (*97*). We used filterByExpr from edgeR v3.30.3 to select 13,398 peaks with more than 10 fragments in 80% of the 15 libraries. The filtered quantification matrix was normalized with the TMM (trimmed mean of M values) method implemented on calcNormFactors (*98*). Counts were then scaled and normalized to log_2_ CPM using limma-voom v3.44.3 (*99*, *100*). The DAC analysis for deceased versus alive at admission and at follow-up was performed with lmFit in limma (*100*). The linear model was adjusted on the covariates age, sex, and FRIP. The log odds of differential accessibility was computed with eBayes (*100*). We estimated Storey’s FDR *q* value in R. Peaks with FDR < 0.05 and absolute log_2_FC > 0.5 were considered significant DACs. To test whether DAC regions were enriched in hospitalized GWAS loci, we used overlapPermTest from regionR (*101*).

### ATAC-seq motif enrichment, GO, and pathway analysis

To test for enrichment of TF binding motifs in DAC regions, we used the findMotifsGenome function implemented in HOMER v4.11 (*102*). DAC regions in deceased versus alive at admission were compared to 50,000 random regions matched by DAC peak mean width and versus nonsignificant regions (FDR > 50%). We considered a motif to be significantly enriched if it presented FDR < 1% versus random and FDR < 10% versus nonsignificant regions in CD14^+^ monocytes. TFs were grouped by family and binding motif similarities after manual curation.

For GO and pathway analyses, we assigned genes to peaks using two approaches. First, peaks were annotated to the genes with a TSS located within 5 kb from the peak boundary using ChIPSeeker v1.24 (*103*). Next, we intersected our peak set with the GeneHancer v5 (2019) dataset using mergeByOverlaps from IRange package v2.22.2 (*104*, *105*). By combining the TSS and GeneHancer approaches, 76.7% of the 13,398 tested peaks were assigned to at least one gene. Enrichment analyses for KEGG, Reactome, and GO biological processes were performed with clusterProfiler v3.16.0 and reactomePA v3.12 (*88*, *106*). For the comparison of open and closed DAC regions at admission, pathways and GO terms with FDR < 1% and at least 10 genes were considered significant. For the follow-up and stratified analyses by motif groups, an FDR < 5% and at least five genes in a pathway were deemed significant.

### Data visualization

To produce genomic tracks for ATAC-seq libraries, we used bamCoverage from DeepTools (*94*) with a scaling factor based on edgeR’s TMM normalization method (*94*). This approach takes into account only fragments in peaks while also normalizing for library size. Next, SparK v2.6.2 was used to calculate the average base pair accessibility and SD per group (*107*). We used removeBatchEffect from limma to calculate the residual log_2_ CPM after regressing out covariates included in the linear models (*100*). Volcano plots were produced with ScatterView from MAGeCKFlute v1.8.0 (*108*). Raincloud, ggpubr, and ggplot2 were used to produce the remaining ATAC-seq plots (*109*, *110*).

### DNA methylation calling and single-nucleotide polymorphism filtering

DNA methylation calling was performed with the GenPipes (v3.1.5-beta) “Methyl Seq” pipeline (*111*). In summary, raw reads were trimmed for quality (quality score ≥ 30) and to remove sequencing adaptors and then aligned to the human genome hg38 reference genome using the Dynamic Read Analysis for Genomics (DRAGEN) epigenome pipeline (v3.4.5), which uses Illumina’s DRAGEN Bio-It Platform with the improved and highly optimized mapping algorithms. All samples had a mean genome coverage of at least 18× (table S8). Duplicate reads were then removed using Picard (https://broadinstitute.github.io/picard/), and methylation calls were extracted using the methylation caller included as part of Bismark (v. 0.18.1) (*112*). CpGs were filtered to remove any position that overlaps with a single-nucleotide polymorphism (SNP) locus to reduce potential bias introduced by genetic variations. SNP loci were extracted from whole-genome sequencing (WGS) libraries from the same patients. GenPipes (v3.1.6-beta) “DNA Seq” pipeline was used to analyze WGS data (*111*). Raw reads were trimmed for quality (quality score ≥ 30) using skewer 0.2.2 and then aligned to the human genome hg38 reference genome using BWA 0.7.17 with standard parameters. Alignment refinement was done using GATK (v3.8) and includes marking duplicate, indel realignment, and base recalibration process. The Haplotype_caller function from GATK (v3.8) was used to call variants (*113*). Every SNP found in at least one sample was used to define the set of SNP loci. On average, 860,127 (3.05%) CpGs were removed from each sample (table S8).

### Detection of DMLs and DMRs

CpG sites with a minimum coverage of two in at least four samples were selected for differential analyses. Methylation raw data were smoothed using a 500-bp smoothing window as a parameter of the BSmooth function implemented in the bsseq Bioconductor package (v. 1.20.0) (*114*). Smoothed methylation levels of the CpG sites located on chromosome 21 of every sample were used to perform a principal components analysis to identify confounding factors. Differential methylation was detected using the R Bioconductor package DSS (v2.32.0) adjusting for patient age and sex (*115*). The model fitting was done by the DSS function DMLfit.multiFactor. Differentially methylated CpG sites (DMLs) were extracted using the DSS function DMLtest.multiFactor. Last, DMLs with *P* value < 0.05 were used to search DMRs using the DSS function callDMR. The direction of the methylation changes (hyper- or hypomethylation) was defined on the basis of the sum of the test statistics of all DMLs within a DMR as provided by the areaStat value in the results of the callDMR function.

### Methylation motif enrichment, GO, and pathway analysis

The analysis of the enrichment of TF motifs in DMRs applied the same procedure as for the looking of TF enrichment in DAC ATAC-seq data. Only DMRs in the deceased versus alive comparison at admission were used to look for TF motifs enrichment. A motif was considered significantly enriched if the FDR was less than 1% when compared to random regions.

To detect GO and pathway enrichments, we categorized DMRs into three groups on the basis of their genomic location: gene body, promoter, and distal DMRs. Gene body DMRs are characterized by DMRs containing at least 10 CpGs located inside a gene. Promoter DMRs correspond to DMRs located in between −5 and +1 kb from a gene TSS. Because of a high number of DMR located in promoter regions, only the top 90% most differently methylated promoter DMRs was conserved for the analysis. Last, distal DMRs were selected by overlapping promoter DMRs with the GeneHancer v5 (2019) dataset using mergeByOverlaps from the IRanges v2.22.2 package and were annotated on the basis of the associated gene (*104*). For each of the three DMR categories, hyper- and hypomethylated DMRs were analyzed separately. GO biological process and KEGG enrichments were detected using the clusterProfiler v3.16.0 from R. Enrichments were considered significant at FDR < 0.05 and if the enrichment is supported by at least five different genes and DMRs (*88*, *106*).

### Data integration and identification of drug candidates

We performed data integration at the pathway and GO term levels. Pathways and GO terms detected by more than one assay with at least 10 genes and FDR < 5% (scRNA, ATAC, or WGBS) were considered for downstream drug discovery. Because of the hierarchical nature of GO terms and Reactome datasets, we selected one term or pathway per hierarchical branch to reduce redundancies for plotting only (full datasets are provided in table S9).

To identify candidate drugs to treat severely ill patients with COVID-19, we first performed an IPA including (i) all DEGs at admission and (ii) filtered for up-regulated DEGs with a corresponding increased DAC peak at admission. Next, to evaluate candidate drugs targeting epigenetically and transcriptionally altered pathways, we used ReactomeFIViz, a tool that provides gene functional interaction networks combined with human curated pathways derived from Reactome. Briefly, to identify drugs interacting with genes and hubs in Reactome pathways, we accessed the DrugCentral database in Cytoscape via ReactomeFIViz (*116*–*118*). The DrugCentral database was created by collecting extensive drug information, including structure, active ingredients, mechanism of action, and target interactions for more than 2000 Food and Drug Adminstration–approved drugs. Genes tagged by at least two assays (scRNA, ATAC, or WGBS) were projected in the Reactome pathway network as shown in fig. S14B. We then used the “Fetch DrugCentral Drugs” function of ReactomeFIViz to identify candidate drugs interacting with nodes of the Reactome pathways tested. In addition, we assessed the KEGG drugs database, which provides a section of drug-pathway interactions, to evaluate candidate drugs in the spliceosome pathways detected in all three tested assays of our study (*119*).

To build a protein network around the targets for candidate drugs, we used OmnipathR protein-protein interaction network and the dbparser packages to access the DrugBank dataset (*120*, *121*). We started by extracting the proteins targeted by the candidate drugs in DrugBank. Next, we selected genes detected by at least two assays (RNA, ATAC, or WGBS) in the Reactome interferon signaling (R-HSA-913531) for nintedanib and mRNA splicing (R-HSA-72172) for tacrolimus and zotatifin as “genes of interest.” We then identified in OmnipathR the shortest protein-protein interaction network between the drug targets and proteins encoded by the genes of interest via intermediary proteins with detectable gene expression in PBMCs.

## Notes

View/request a protocol for this paper from *Bio-protocol*.
