# Supplementary material for: A systems biology approach identifies candidate drugs to reduce mortality in severely ill patients with COVID-19
Source: Sci Adv. 2022 Jun 1;8(22):eabm2510. doi: 10.1126/sciadv.abm2510 (PMC9159580; doi:10.1126/sciadv.abm2510)
Supplement: Supplementary file 1 — Figs. S1 to S15 [file sciadv.abm2510_sm.pdf]

Science Advances
AAAS

Supplementary Materials for

A systems biology approach identifies candidate drugs to reduce mortality in severely ill patients with COVID-19

Vinicius M. Fava *et al.*

Corresponding author: David Langlais, david.langlais@mcgill.ca; Erwin Schurr, erwin.schurr@mcgill.ca

Sci. Adv. **8**, eabm2510 (2022)
DOI: 10.1126/sciadv.abm2510

The PDF file includes:

Figs. S1 to S15
Legends for tables S1 to S9

Other Supplementary Materials in this manuscript includes the following:

Tables S1 to S9

SUPPLEMENTAL MATERIAL

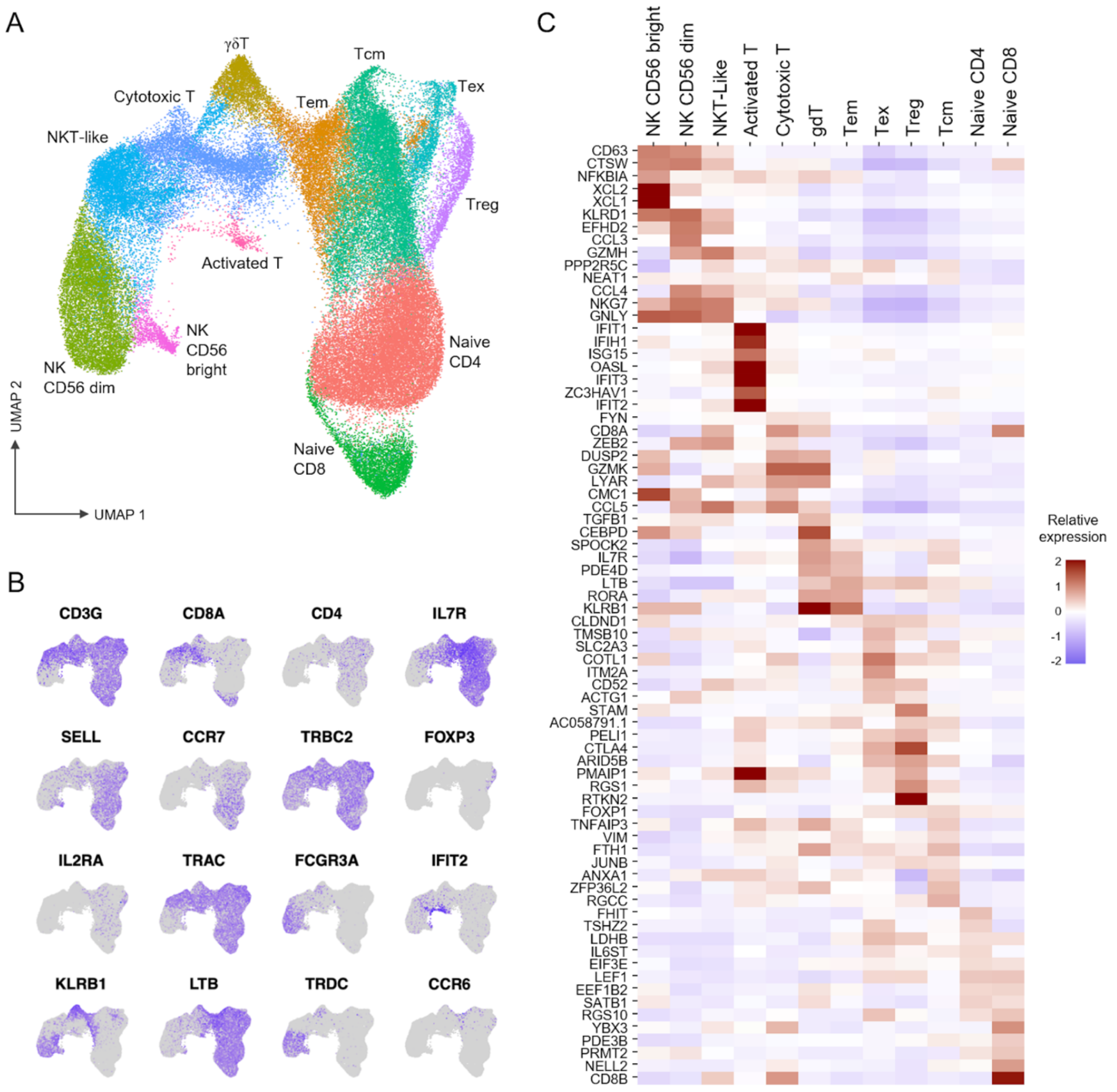

Figure S1. Sub-population composition of T and NK cells from COVID-19 patients.

(A) UMAP visualization for the subclustering of T and NK cells selected PBMC of 7 COVID-19 patients (18 samples obtained at days 0, 5 and 15 post admission) and 6 healthy controls. Twelve major sub-populations were identified. **(B)** UMAP visualization of sixteen major T cell marker genes used to validate sub-populations annotation. **(C)** Heatmap representation of the normalized expression in each individual T-cell subpopulation for the top 74 genes identified as sub-population specific markers using the Seurat 'FindAllMarkers' function.

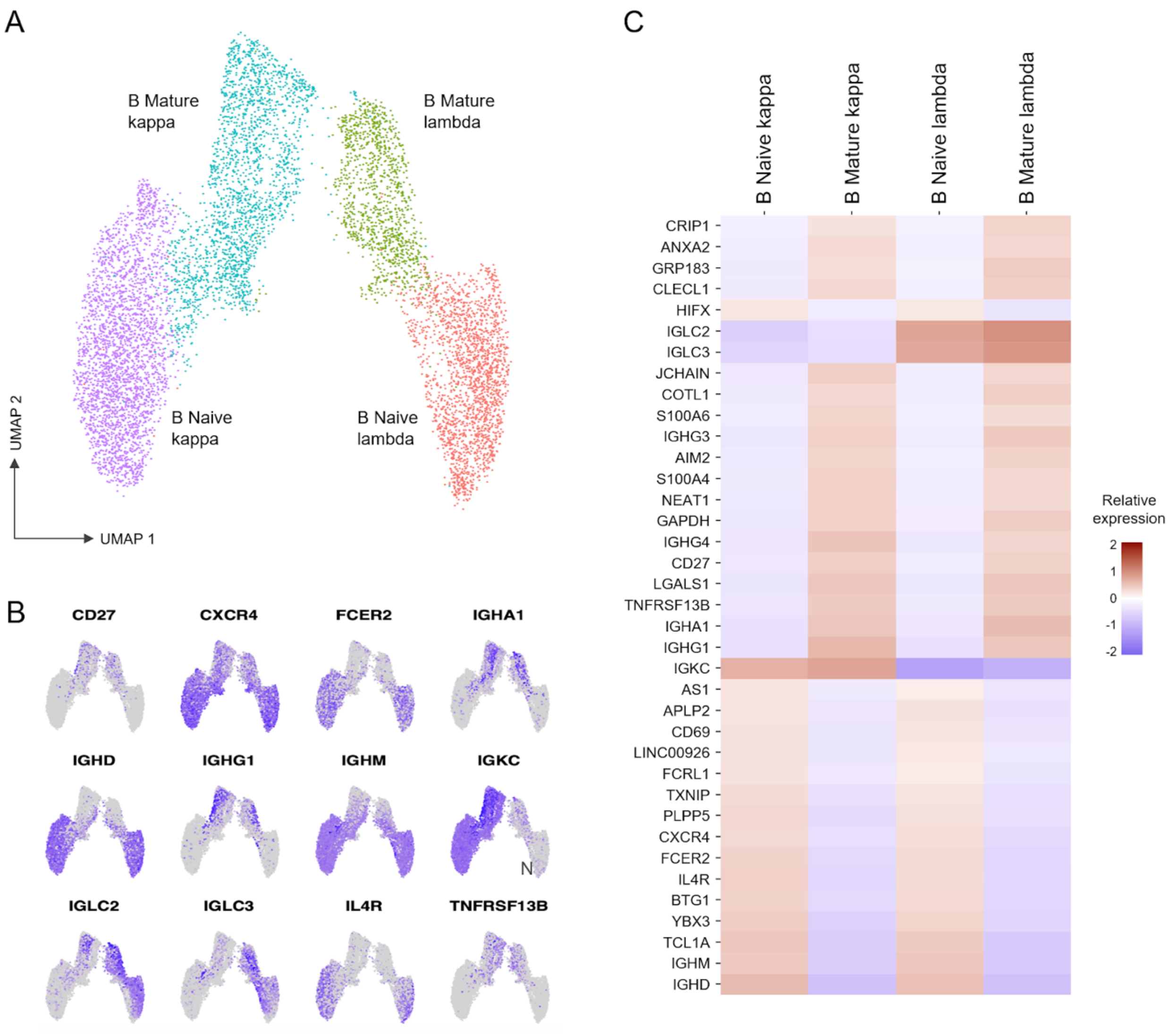

Figure S2. Sub-population composition of B cells from COVID-19 patients.

(A) UMAP visualization of B cell sub-populations selected from 7 COVID-19 patients (18 samples obtained at days 0, 5 and 15 of admission) and 6 healthy controls. Four major sub-populations are identified. **(B)** UMAP visualization of twelve major B-cell marker genes used to validate sub-population annotation. **(C)** Heatmap representation of the normalized expression in each individual B-cell subpopulation for the top 37 genes identified as sub-population specific markers using the Seurat 'FindAllMarkers' function.

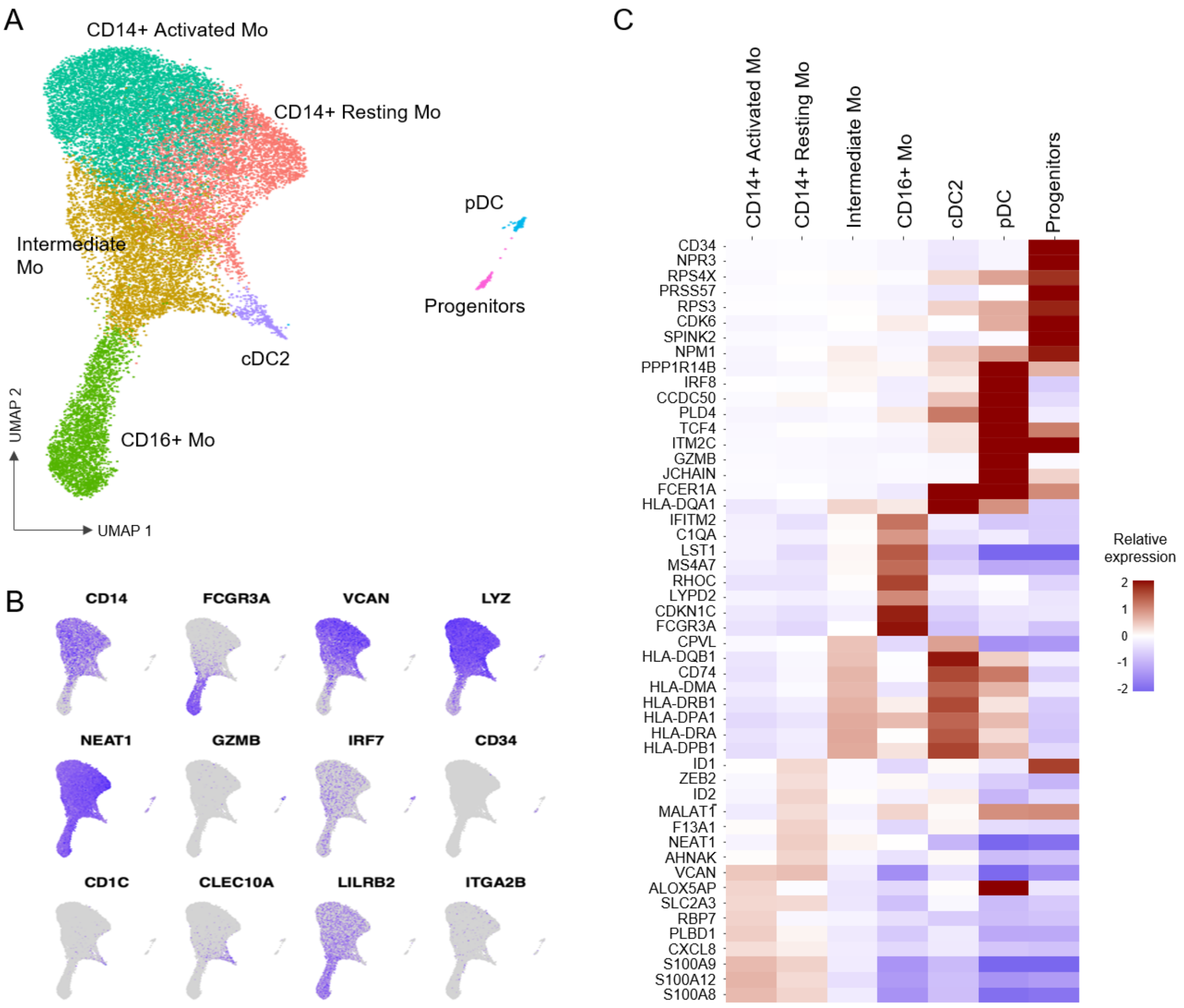

Figure S3. Sub-population composition of Myeloid cells from COVID-19 patients.

(A) UMAP visualization of Myeloid cells subclustering selected from PBMC of 7 COVID-19 patients (18 samples obtained at days 0, 5 and 15 of admission) and 6 healthy controls. Seven major sub-populations were identified. **(B)** UMAP visualization of twelve marker genes used to validate sub-population annotation. **(C)** Heatmap representation of the normalized expression in each Myeloid cell subpopulation for the top 50 genes identified as sub-population specific markers using the Seurat 'FindAllMarkers' function.

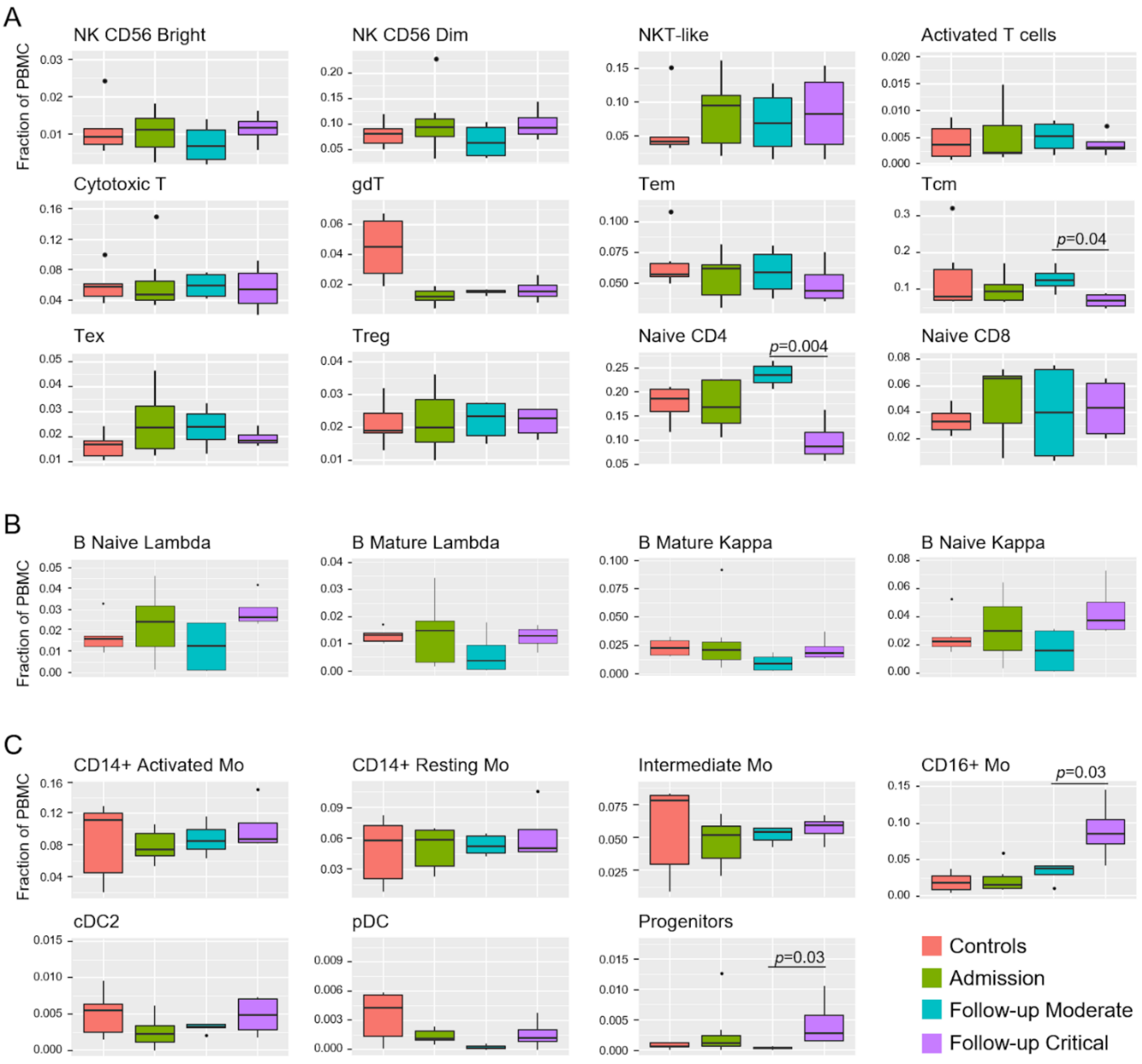

Figure S4. PBMC sub-population cell proportions

Box and whisker plots for the proportion of cells for each subpopulation of T cells **(A)**, B cells **(B)** and Myeloid cells **(C)** for samples in each clinical group; expressed as % of total PBMC. Lineage proportions at days 5 and 15 of COVID-19 patients classified based on the disease OS and grouped as Moderate(cyan) or Critical(purple). Corresponding values from healthy controls (red) and from all COVID-19 patients at admission (green) are provided as reference groups. Q-values for pairwise t-test between Critical and Moderate comparisons are provided when significant.

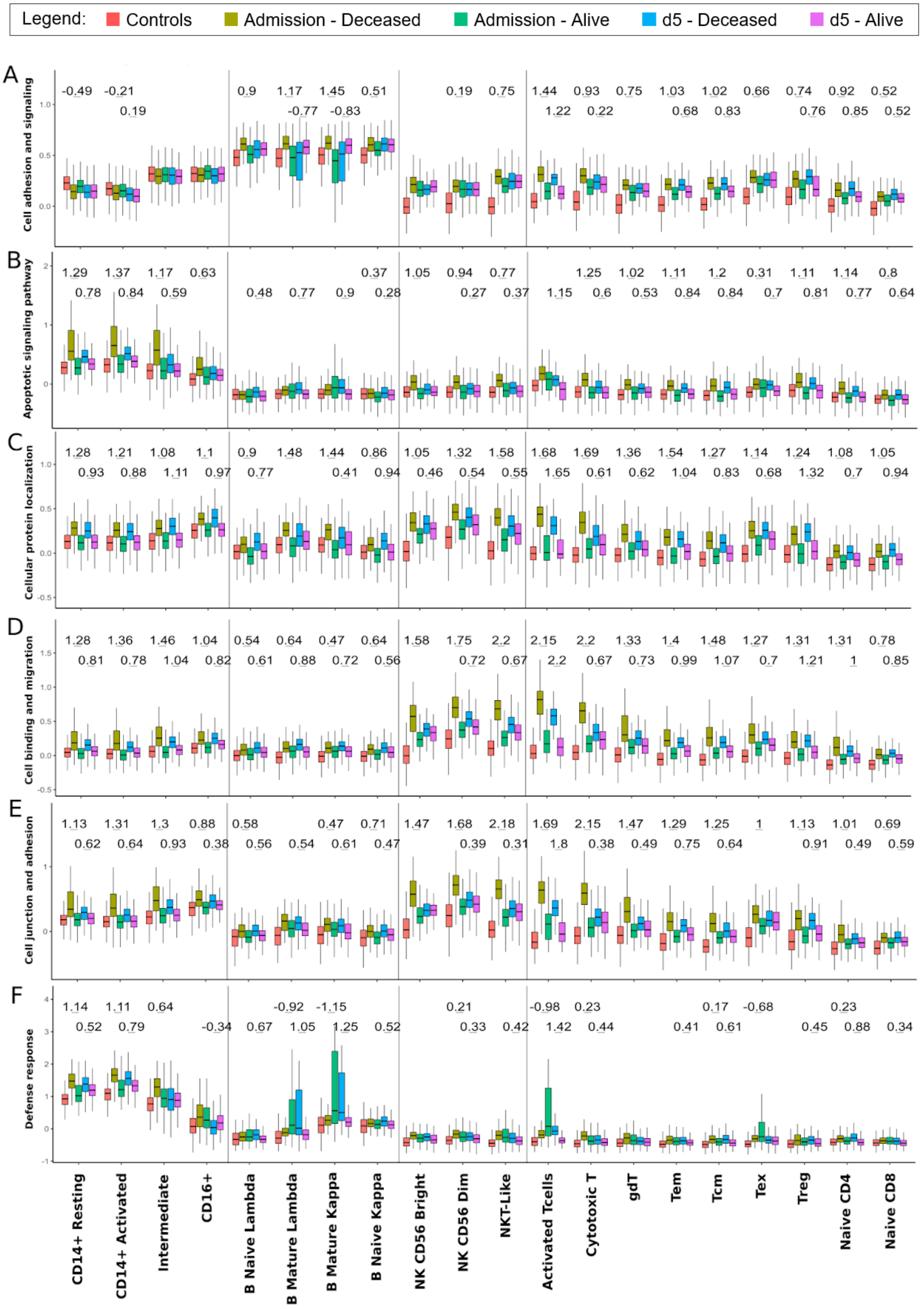

Legend: Controls Admission - Deceased Admission - Alive d5 - Deceased d5 - Alive
A
Cell adhesion and signaling
-0.49 -0.21 0.9 1.17 1.45 0.51 0.19 0.75 1.44 0.93 0.75 1.03 1.02 0.66 0.74 0.92 0.52
0.19 -0.77 -0.83 1.22 0.22 0.68 0.83 0.76 0.85 0.52
B
Apoptotic signaling pathway
1.29 1.37 1.17 0.63 0.37 1.05 0.94 0.77 1.25 1.02 1.11 1.2 0.31 1.11 1.14 0.8
0.78 0.84 0.59 0.48 0.77 0.9 0.28 0.27 0.37 1.15 0.6 0.53 0.84 0.84 0.7 0.81 0.77 0.64
C
Cellular protein localization
1.28 1.21 1.08 1.1 0.9 1.48 1.44 0.86 1.05 1.32 1.58 1.68 1.69 1.36 1.54 1.27 1.14 1.24 1.08 1.05
0.93 0.88 1.11 0.97 0.77 0.41 0.94 0.46 0.54 0.55 1.65 0.61 0.62 1.04 0.83 0.68 1.32 0.7 0.94
D
Cell binding and migration
1.28 1.36 1.46 1.04 0.54 0.64 0.47 0.64 1.58 1.75 2.2 2.15 2.2 1.33 1.4 1.48 1.27 1.31 1.31 0.78
0.81 0.78 1.04 0.82 0.61 0.88 0.72 0.56 0.72 0.67 2.2 0.67 0.73 0.99 1.07 0.7 1.21 1 0.85
E
Cell junction and adhesion
1.13 1.31 1.3 0.88 0.58 0.47 0.71 1.47 1.68 2.18 1.69 2.15 1.47 1.29 1.25 1 1.13 1.01 0.69
0.62 0.64 0.93 0.38 0.56 0.54 0.61 0.47 0.39 0.31 1.8 0.38 0.49 0.75 0.64 0.91 0.49 0.59
F
Defense response
1.14 1.11 0.64 -0.92 -1.15 0.21 -0.98 0.23 0.17 -0.68 0.23
0.52 0.79 -0.34 0.67 1.05 1.25 0.52 0.33 0.42 1.42 0.44 0.41 0.61 0.45 0.88 0.34
CD14+ Resting
CD14+ Activated
Intermediate
CD16+
B Naive Lambda
B Mature Lambda
B Mature Kappa
B Naive Kappa
NK CD56 Bright
NK CD56 Dim
NKT-Like
Activated Tcells
Cytotoxic T
gdT
Tem
Tcm
Tex
Treg
Naive CD4
Naive CD8

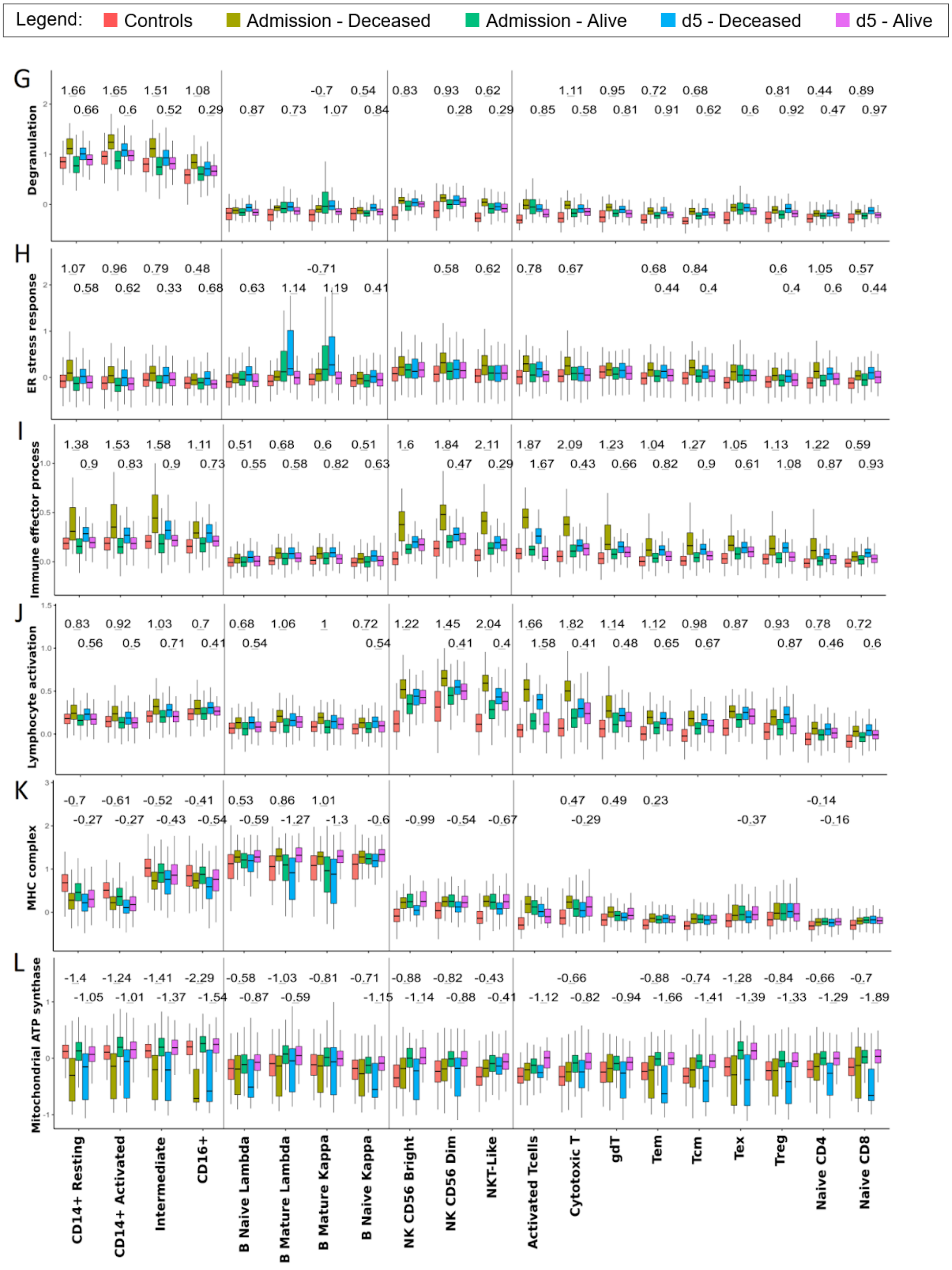
Legend: Controls Admission - Deceased Admission - Alive d5 - Deceased d5 - Alive
G
Degranulation
1.66 1.65 1.51 1.08 -0.7 0.54 0.83 0.93 0.62 1.11 0.95 0.72 0.68 0.81 0.44 0.89
0.66 0.6 0.52 0.29 0.87 0.73 1.07 0.84 0.28 0.29 0.85 0.58 0.81 0.91 0.62 0.6 0.92 0.47 0.97
H
ER stress response
1.07 0.96 0.79 0.48 -0.71 0.58 0.62 0.78 0.67 0.68 0.84 0.6 1.05 0.57
0.58 0.62 0.33 0.68 0.63 1.14 1.19 0.41 0.44 0.4 0.4 0.6 0.44
I
Immune effector process
1.38 1.53 1.58 1.11 0.51 0.68 0.6 0.51 1.6 1.84 2.11 1.87 2.09 1.23 1.04 1.27 1.05 1.13 1.22 0.59
0.9 0.83 0.9 0.73 0.55 0.58 0.82 0.63 0.47 0.29 1.67 0.43 0.66 0.82 0.9 0.61 1.08 0.87 0.93
J
Lymphocyte activation
0.83 0.92 1.03 0.7 0.68 1.06 1 0.72 1.22 1.45 2.04 1.66 1.82 1.14 1.12 0.98 0.87 0.93 0.78 0.72
0.56 0.5 0.71 0.41 0.54 0.54 0.41 0.4 1.58 0.41 0.48 0.65 0.67 0.87 0.46 0.6
K
MHC complex
-0.7 -0.61 -0.52 -0.41 0.53 0.86 1.01 0.47 0.49 0.23 -0.14
-0.27 -0.27 -0.43 -0.54 -0.59 -1.27 -1.3 -0.6 -0.99 -0.54 -0.67 -0.29 -0.37 -0.16
L
Mitochondrial ATP synthase
-1.4 -1.24 -1.41 -2.29 -0.58 -1.03 -0.81 -0.71 -0.88 -0.82 -0.43 -0.66 -0.88 -0.74 -1.28 -0.84 -0.66 -0.7
-1.05 -1.01 -1.37 -1.54 -0.87 -0.59 -1.15 -1.14 -0.88 -0.41 -1.12 -0.82 -0.94 -1.66 -1.41 -1.39 -1.33 -1.29 -1.89
CD14+ Resting
CD14+ Activated
Intermediate
CD16+
B Naive Lambda
B Mature Lambda
B Mature Kappa
B Naive Kappa
NK CD56 Bright
NK CD56 Dim
NKT-Like
Activated Tcells
Cytotoxic T
gdT
Tem
Tcm
Tex
Treg
Naive CD4
Naive CD8

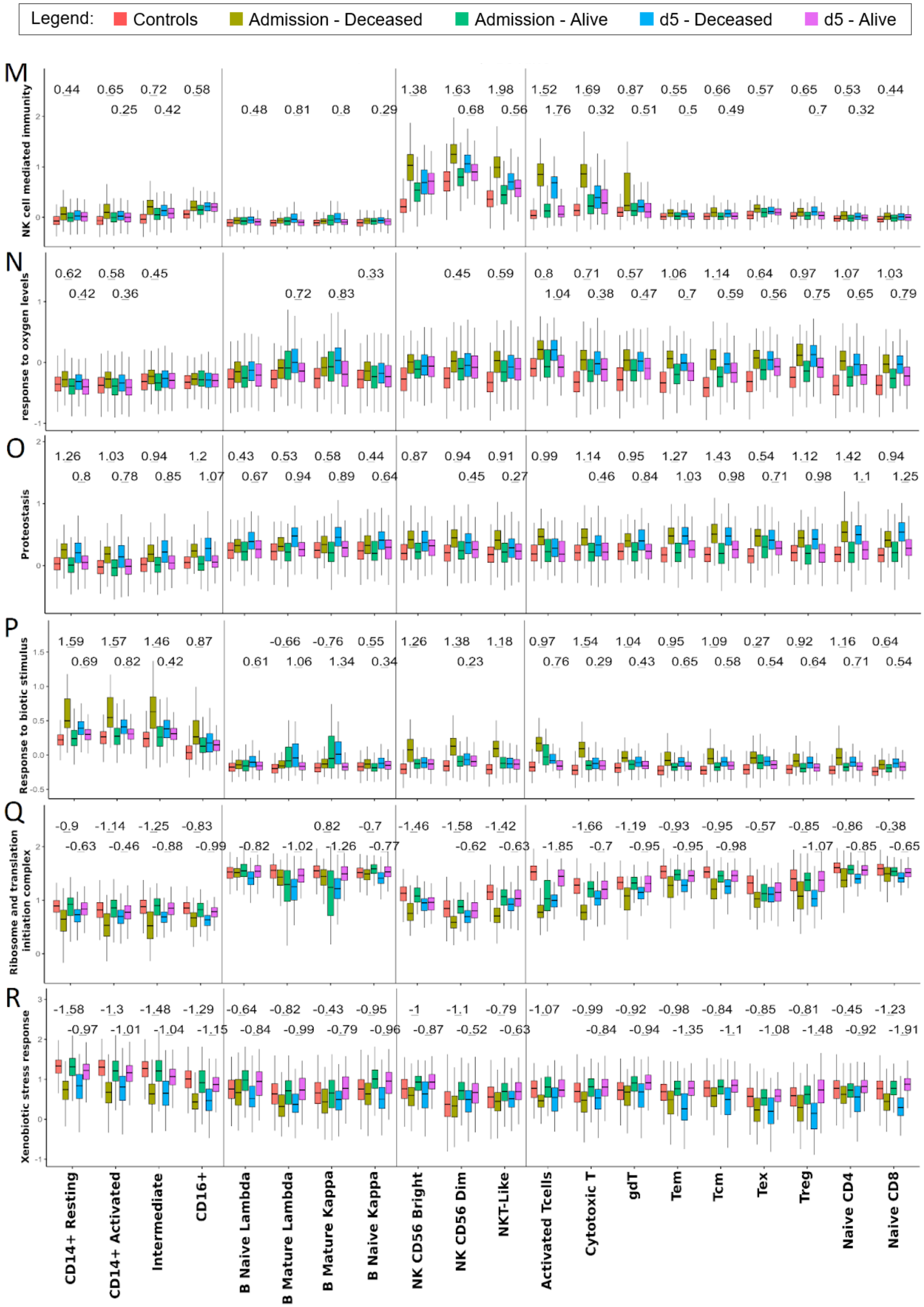
Legend: Controls Admission - Deceased Admission - Alive d5 - Deceased d5 - Alive
M
NK cell mediated immunity
0.44 0.65 0.72 0.58 1.38 1.63 1.98 1.52 1.69 0.87 0.55 0.66 0.57 0.65 0.53 0.44
0.25 0.42 0.48 0.81 0.8 0.29 0.68 0.56 1.76 0.32 0.51 0.5 0.49 0.7 0.32
N
response to oxygen levels
0.62 0.58 0.45 0.33 0.45 0.59 0.8 0.71 0.57 1.06 1.14 0.64 0.97 1.07 1.03
0.42 0.36 0.72 0.83 1.04 0.38 0.47 0.7 0.59 0.56 0.75 0.65 0.79
O
Proteostasis
1.26 1.03 0.94 1.2 0.43 0.53 0.58 0.44 0.87 0.94 0.91 0.99 1.14 0.95 1.27 1.43 0.54 1.12 1.42 0.94
0.8 0.78 0.85 1.07 0.67 0.94 0.89 0.64 0.45 0.27 0.46 0.84 1.03 0.98 0.71 0.98 1.1 1.25
P
Response to biotic stimulus
1.59 1.57 1.46 0.87 -0.66 -0.76 0.55 1.26 1.38 1.18 0.97 1.54 1.04 0.95 1.09 0.27 0.92 1.16 0.64
0.69 0.82 0.42 0.61 1.06 1.34 0.34 0.23 0.76 0.29 0.43 0.65 0.58 0.54 0.64 0.71 0.54
Q
Ribosome and translation initiation complex
-0.9 -1.14 -1.25 -0.83 0.82 -0.7 -1.46 -1.58 -1.42 -1.66 -1.19 -0.93 -0.95 -0.57 -0.85 -0.86 -0.38
-0.63 -0.46 -0.88 -0.99 -0.82 -1.02 -1.26 -0.77 -0.62 -0.63 -1.85 -0.7 -0.95 -0.95 -0.98 -1.07 -0.85 -0.65
R
Xenobiotic stress response
-1.58 -1.3 -1.48 -1.29 -0.64 -0.82 -0.43 -0.95 -1 -1.1 -0.79 -1.07 -0.99 -0.92 -0.98 -0.84 -0.85 -0.81 -0.45 -1.23
-0.97 -1.01 -1.04 -1.15 -0.84 -0.99 -0.79 -0.96 -0.87 -0.52 -0.63 -0.84 -0.94 -1.35 -1.1 -1.08 -1.48 -0.92 -1.91
CD14+ Resting
CD14+ Activated
Intermediate
CD16+
B Naive Lambda
B Mature Lambda
B Mature Kappa
B Naive Kappa
NK CD56 Bright
NK CD56 Dim
NKT-Like
Activated Tcells
Cytotoxic T
gdT
Tem
Tcm
Tex
Treg
Naive CD4
Naive CD8

Figure S5. Evolution of the transcriptional expression for enriched GO-terms.

Differentially expressed genes contributing to GO term enrichment (indicated on the left axis and displayed in Fig. 4A) have been used to generate a Module Score representing the overall transcriptional expression level for the specific category. Corresponding cell populations are listed at the bottom of the graph. Module scores have been computed for the Day 0 and Day 5 time-points for each cell population and based on "Deceased" and "Alive" COVID-19 patient outcomes separately. Control samples from healthy subjects are included as reference. Sample groups are coloured according to the key on top of the graph. The significance of the gene expression differences between "Deceased vs Alive" COVID-19 patients was tested by comparing the corresponding Module Score distribution in every group of cells using a wilcoxon rank-test approach (q-value <= 0.05). To provide a sample-size free evaluation of the difference in expression, Cohen's d effect size estimation is shown above each significant variation observed.

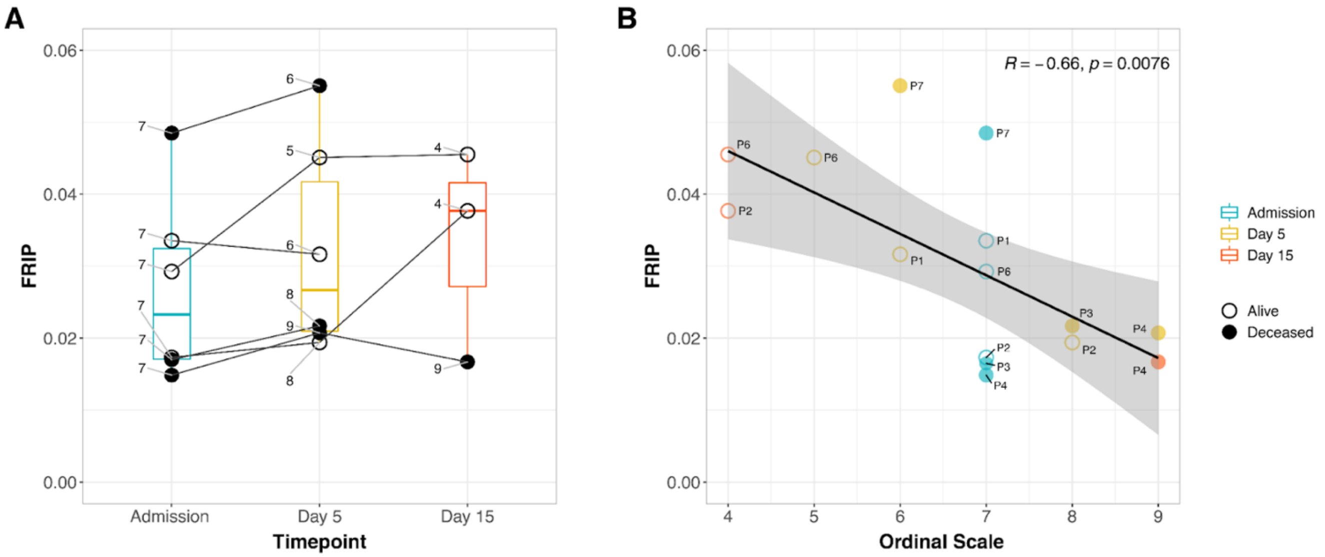

Figure S6. COVID-19 WHO clinical ordinal scale correlates with chromatin stability of CD14+ monocytes.

(A) The fraction of reads in peaks (FRIP) for CD14+ monocytes, a surrogate for chromatin stability, was plotted against the date of PBMC collection. The WHO ordinal scale for COVID-19 is shown for all patients at each timepoint. Horizontal lines link the same patient at different timepoints. **(B)** Correlation of the FRIP with WHO clinical scale score for COVID-19. Samples are labeled according to the donor ID, day of collection and patient outcome.

716 More close peaks in deceased at Admission

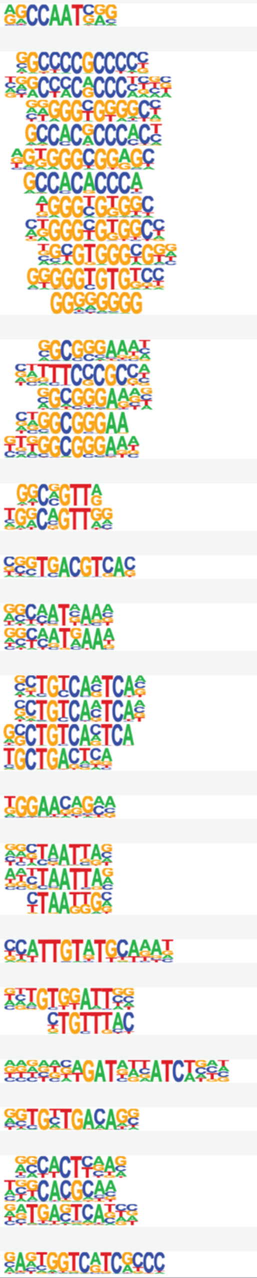

Name	MOTIF	# of DAC with Motif	% of DAC with Motif	# random peaks with Motif	% of random peaks with Motif	# peaks at FDR > 50% with Motif	% peaks at FDR > 50% with Motif	*p*-value	FDR q-value
**Group 1 - NFY**									
NFY		282	49.3%	12676	27.4%	1356	34.9%	1.0E-28	< 5.0E-05
**Group 2 - Sp1-like/KLF**									
Sp1		297	51.9%	14991	32.4%	1592	40.9%	1.0E-21	< 5.0E-05
KLF3		308	53.9%	16188	34.9%	1642	42.2%	1.0E-19	< 5.0E-05
KLF1		404	70.6%	24860	53.7%	2318	59.6%	1.0E-16	< 5.0E-05
Klf9		238	41.6%	13083	28.2%	1256	32.3%	1.0E-11	< 5.0E-05
Sp5		409	71.5%	27005	58.3%	2488	64.0%	1.0E-10	< 5.0E-05
Klf4		189	33.0%	10073	21.7%	968	24.9%	1.0E-09	< 5.0E-05
KLF5		419	73.3%	29349	63.4%	2612	67.2%	1.0E-06	< 5.0E-05
KLF6		389	68.0%	26803	57.9%	2389	61.4%	1.0E-06	< 5.0E-05
Egr2		136	23.8%	7583	16.4%	671	17.3%	1.0E-05	< 5.0E-05
KLF10		193	33.7%	12843	27.7%	1100	28.3%	1.0E-03	5.6E-03
Maz		412	72.0%	30442	65.7%	2566	66.0%	1.0E-03	4.5E-03
**Group 3 - E2F**									
E2F4		273	47.7%	17136	37.0%	1629	41.9%	1.0E-07	< 5.0E-05
E2F7		101	17.7%	5881	12.7%	556	14.3%	1.0E-03	2.8E-03
E2F6		274	47.9%	18963	40.9%	1642	42.2%	1.0E-03	3.0E-03
E2F1		173	30.2%	11181	24.1%	996	25.6%	1.0E-03	3.3E-03
E2F3		298	52.1%	20918	45.2%	1818	46.8%	1.0E-03	3.3E-03
**Group 4 - MYB**									
MYB		369	64.5%	23351	50.4%	2064	53.1%	1.0E-11	< 5.0E-05
AMYB		302	52.8%	19292	41.6%	1815	46.7%	1.0E-07	< 5.0E-05
**Group 5 - CRE**									
CRE		114	19.9%	5163	11.1%	599	15.4%	1.0E-09	< 5.0E-05
**Group 6 - HOX**									
Hoxa9		362	63.3%	24219	52.3%	2058	52.9%	1.0E-07	< 5.0E-05
Hoxd10		190	33.2%	11890	25.7%	1069	27.5%	1.0E-04	3.0E-04
**Group 7 - PBX**									
Pknox1		75	13.1%	3567	7.7%	308	7.9%	1.0E-05	1.0E-04
Pbx3		66	11.5%	3201	6.9%	263	6.8%	1.0E-04	4.0E-04
PBX1		30	5.2%	1072	2.3%	84	2.2%	1.0E-04	4.0E-04
MafA		165	28.9%	10200	22.0%	896	23.0%	1.0E-04	7.0E-04
**Group 8 - ZNF189**									
ZNF189		203	35.5%	12778	27.6%	1056	27.2%	1.0E-04	3.0E-04
**Group 9 - LIM homeobox**									
En1		271	47.4%	18020	38.9%	1600	41.1%	1.0E-04	3.0E-04
Isl1		280	49.0%	18856	40.7%	1596	41.0%	1.0E-04	4.0E-04
Lhx3		230	40.2%	15145	32.7%	1241	31.9%	1.0E-04	8.0E-04
**Group 10 - Oct4:Sox17**									
Oct4:Sox17		33	5.8%	1222	2.6%	65	1.7%	1.0E-04	3.0E-04
**Group 11 - FOX**									
Foxh1		115	20.1%	6695	14.5%	556	14.3%	1.0E-03	1.2E-03
Foxo1		288	50.4%	19992	43.2%	1734	44.6%	1.0E-03	2.3E-03
**Group 12 - GATA**									
GATA		32	5.6%	1394	3.0%	120	3.1%	1.0E-03	4.6E-03
**Group 13 - TBX**									
Tbx20		56	9.8%	2936	6.3%	258	6.6%	1.0E-03	5.8E-03
**Group 14 - NKX**									
Arnt:Ahr		194	33.9%	12608	27.2%	1154	29.7%	1.0E-03	1.8E-03
Nkx2.2		336	58.7%	24193	52.2%	1946	50.0%	1.0E-03	5.9E-03
Nkx2.1		404	70.6%	29952	64.7%	2395	61.6%	1.0E-02	7.9E-03
**Group 15 - ZNF669**									
ZNF669		45	7.9%	2290	4.9%	170	4.4%	1.0E-02	9.4E-03

243 More open peaks in deceased at Admission

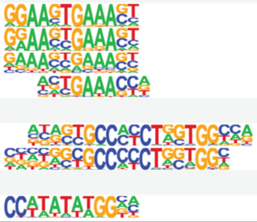

Name	MOTIF	# of DAC with Motif	% of DAC with Motif	# random peaks with Motif	% of random peaks with Motif	# peaks at FDR > 50% with Motif	% peaks at FDR > 50% with Motif	*p*-value	FDR q-value
**Group 16 - IRF**									
PU.1:IRF8		35	16.6%	2597	5.2%	346	9.8%	1.0E-08	< 5.0E-05
IRF8		43	20.4%	4285	8.7%	541	15.3%	1.0E-06	< 5.0E-05
IRF2		17	8.1%	1279	2.6%	193	5.5%	1.0E-04	1.5E-03
IRF4		37	17.5%	4711	9.5%	451	12.8%	1.0E-03	3.8E-03
**Group 17 - CTCF**									
CTCF		30	14.2%	3261	6.6%	324	9.2%	1.0E-04	1.8E-03
BORIS		53	25.1%	7884	15.9%	603	17.1%	1.0E-03	5.9E-03
**Group 18 - MADS**									
CArG		29	13.7%	3423	6.9%	236	6.7%	1.0E-03	5.2E-03

Figure S7. Motif homology groping for the 46 transcription factors enriched in DAC regions of deceased patients at Admission.

The 46 transcription factors significantly enriched in DAC regions of deceased patients at FDR <1% are shown with their respective motif binding sequences in each row. Transcription factors were grouped based on the sequence homology of their binding motif. The number and percentage of peaks with motifs corresponding to the heatmap in Fig. 4D is shown for DAC, random peaks, and peaks at FDR > 50% in the center columns. Uncorrected *p*-value and the FDR q-value for the contrast between DAC and random regions is given in the last two columns.

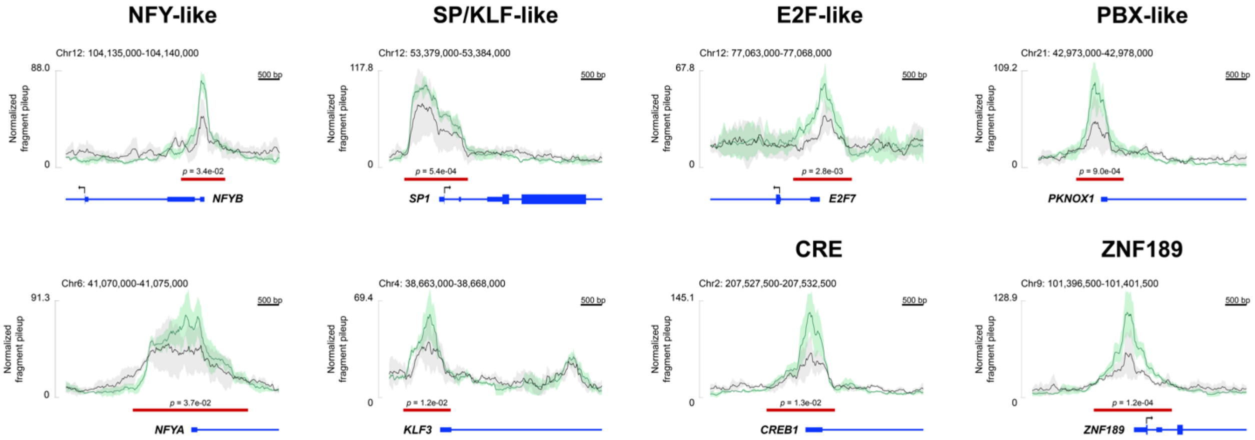

Figure S8. Chromatin accessibility at promoters of transcription factors with binding motifs enriched in repressed DAC regions of deceased COVID-19 patients.

The mean chromatin accessibility at admission for patients retrospectively classified as Deceased or Alive are shown as black and green lines, respectively. Shades indicate the standard deviation of the mean for each group. At the bottom, a red bar denotes the regions contrasted in the “Deceased vs Alive” comparison with its corresponding *p* value.

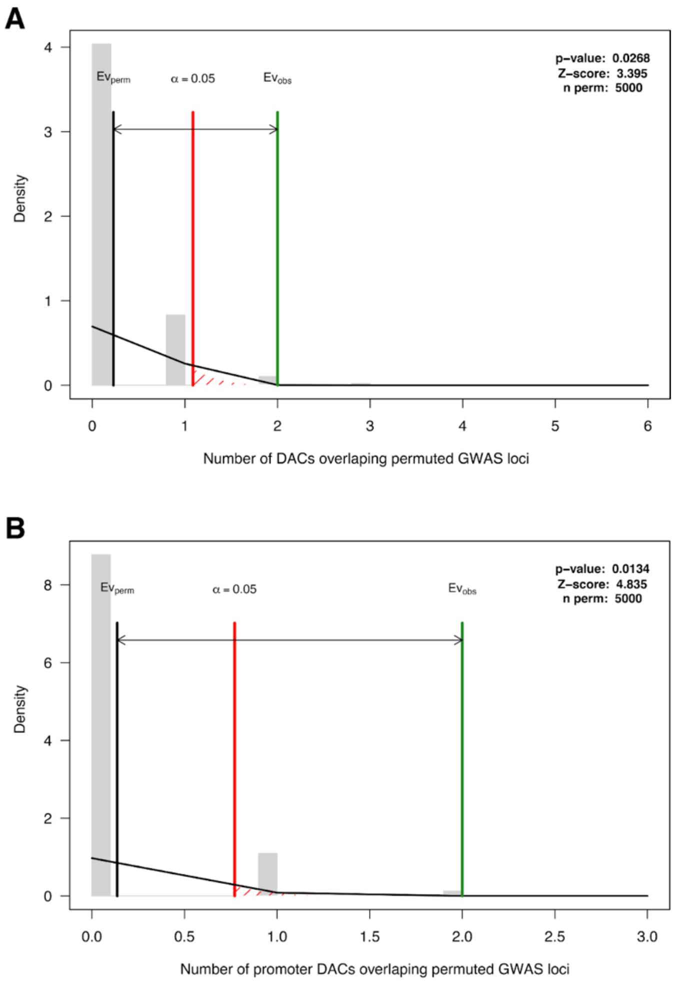

Figure S9. Non-random overlap between DAC for "Deceased" patient monocytes at admission with hospitalized COVID-19 GWAS loci.

The evaluation of a non-random overlap between DAC regions with five significant/suggestive GWAS loci for hospitalized COVDI-19 patients was performed using a permutation test. Genomic regions of equal length to the five GWAS loci were permuted across the human genome while the genomic position for all 959 "Deceased vs Alive" DAC regions for monocytes at admission **(A)**, and for the subset of 481 DAC located in promoter regions **(B)**. The bar plots summarize the number of permuted GWAS loci overlapping a DAC region in the x-axis with the grey bars representing the density plotted in the y-axis. The black vertical line (Ev_{perm}) indicates the average number of permuted GWAS loci encompassing a DAC; the red line represents the 95 percentile of the distribution; and the green line (Ev_{obs}) highlights the number of GWAS loci with a DAC in our study.

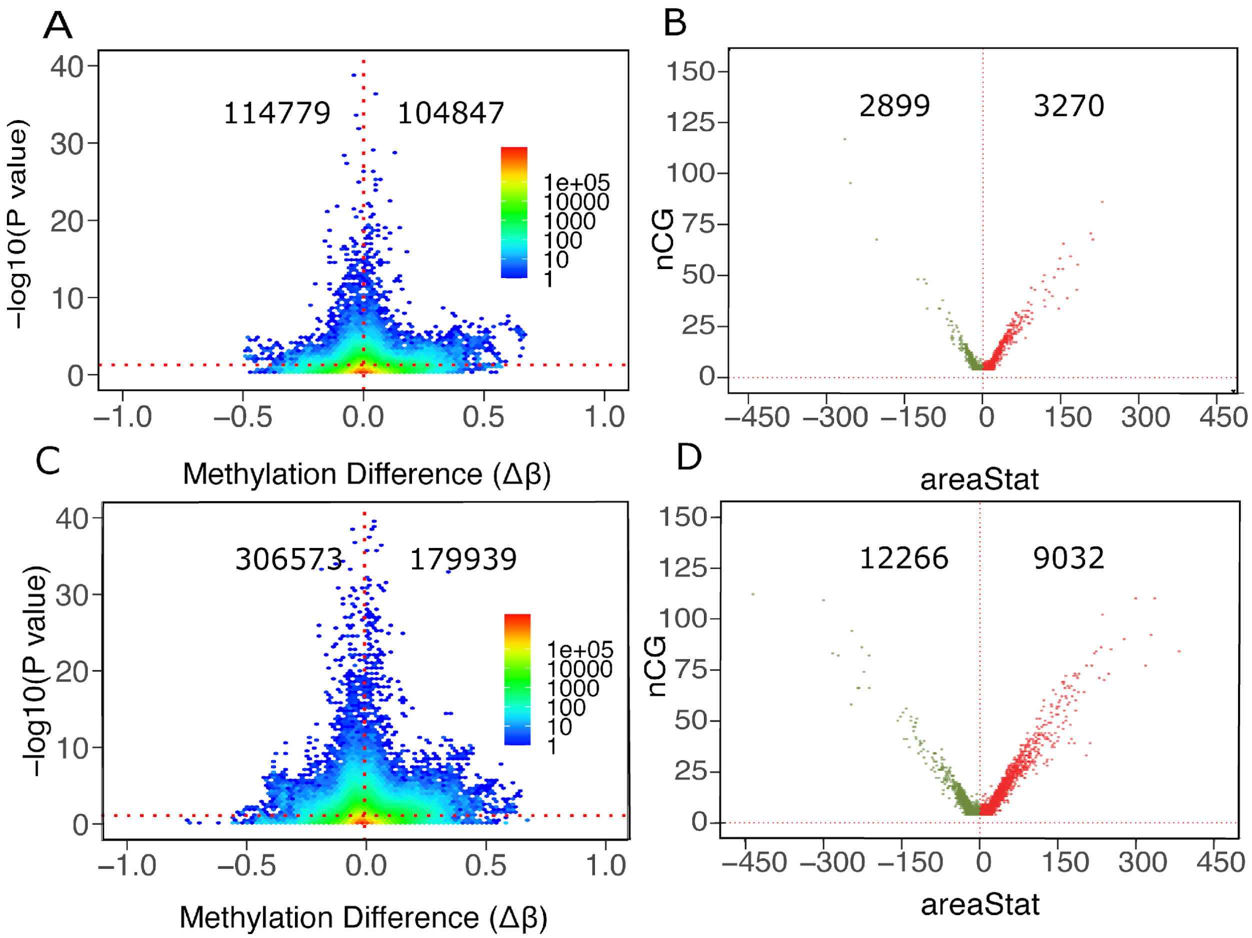

Figure S10. Differential DNA methylation among COVID-19 patients.

(A) A total of 219,626 DMLs were identified for the "Deceased vs Alive" patient comparison at admission. Hyper (Δβ > 0) and hypomethylated (Δβ <=0) CpGs were plotted against their corresponding *p* values. The color gradient indicates the density of CpGs. **(B)** AreasStats of hyper (areaStat > 0) and hypomethylated (areaStat < 0) regions were plotted against the number of CpGs located in the DMR for "Deceased vs Alive" patients at admission (red indicates hypermethylated DMRs and olive green indicates hypomethylated DMRs). **(C)** A total of 486,512 DMLs were detected for the "Deceased vs Alive" patient groups at follow-up. Hyper and hypomethylated CpGs were plotted against their corresponding *p* values. **(D)** AreasStats of hyper (areaStat > 0) and hypomethylated (areaStat < 0) regions were plotted against the number of CpGs located in the DMR for "Deceased vs Alive" patients at follow-up.

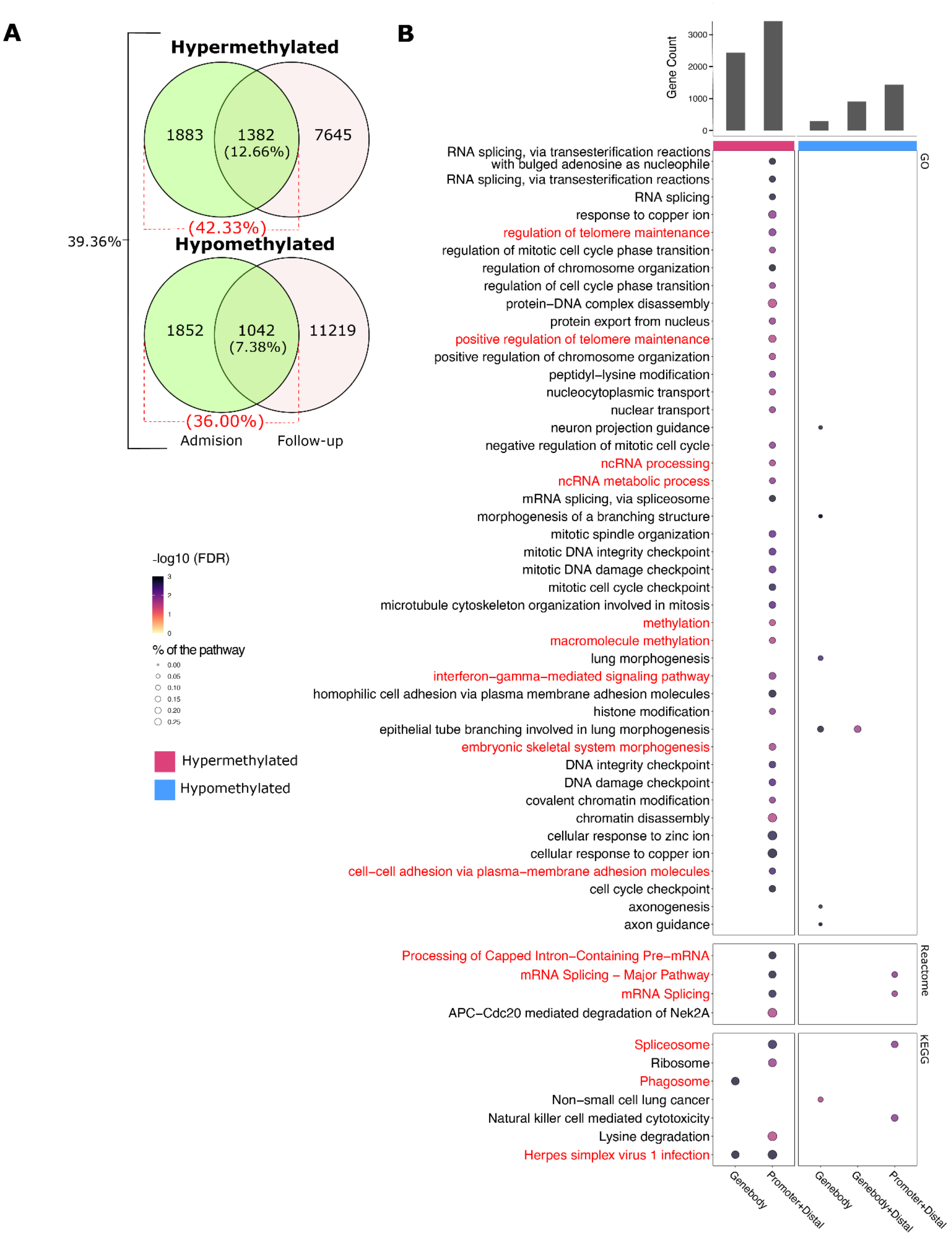

Figure S11: Comparison of monocytes DNA methylation at admission and follow-up. **(A)** Number and percentages of overlapping DMRs between monocytes of patients classified on the disease outcome ("Deceased vs Alive") at the time of admission and follow-up. Overall intersecting percentages for both hyper and hypomethylated DMRs are shown on the left. **(B)** Gene ontology and pathway

enrichment analyses for DMRs at follow-up. Each bubble indicates an ontology or pathway for each of the three tested databases: KEGG and Reactome pathways, and Gene Ontology. The bubble size represents the percentage of genes with a corresponding significant DMR at FDR <5% and shades represent the -log10(FDR). The total number of peaks with assigned genes per group is shown in the bar plot on the right. Pathways also enriched at admission are colored in red.

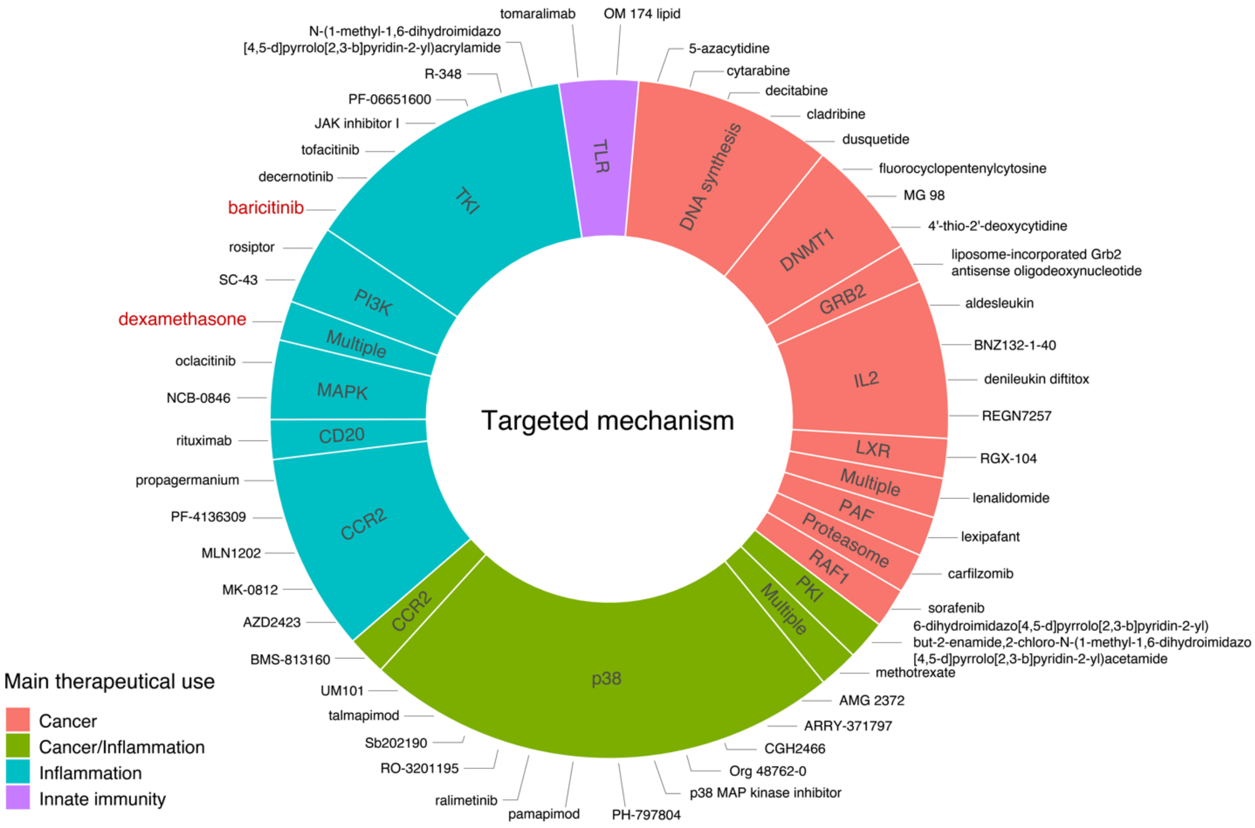

Figure S12. Candidate drugs identified by Ingenuity Pathway Analysis (IPA) with upregulated DEG with increased DAC for Deceased patients at ICU admission. Drugs identified by the Ingenuity Pathway Analysis (IPA) are shown in a pie chart. A total of 50 drugs could be divided in 16 inhibitor/target classes while three drugs presented multiple therapeutic mechanisms. The classes are identified in the pie slices with the colours indicating the main therapeutic use of the candidate drug. Dexamethasone and baricitinib, two drugs currently recommended to treat severe COVID-19, are highlighted in bold red.

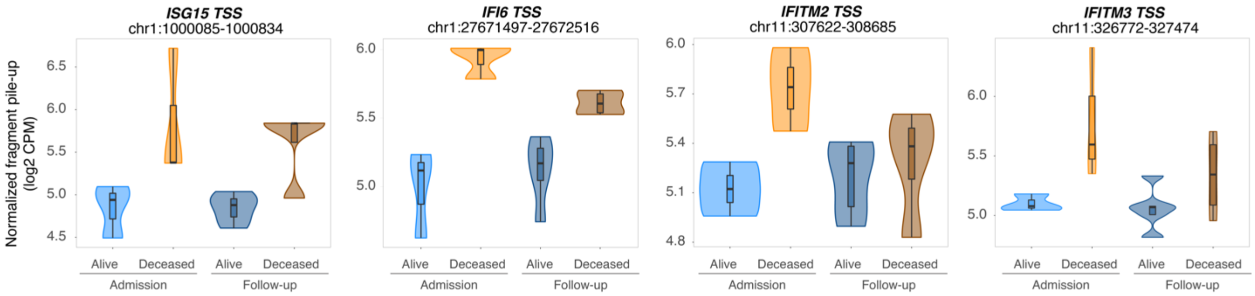

Figure S13: Genes in type-1 interferon signaling GO term show increased promoter accessibility. Box plots with normalized quantification of pileup fragments in peaks at promoter regions for "Deceased" and "Alive" are shown as log2 count per million (CPM) for patient groups at admission and follow-up.

A

Study	Wilk *et al.*	Current study	Schulte-Schrepping *et al.*
Technology	Seq-Well	10X 3'RNA v3.1	10X 3'RNA v3.1
Samples	(PBMC + whole blood)	frozen PBMC	fresh PBMC
Cohort	2 Ctl, 4 Dec., 3 Alive	6 Ctl, 3 Dec., 4 Alive	0 Ctl, 3 Dec., 7 Alive
Patient sex	1 F, 6 M	3 F, 4 M	4 F, 6 M
Retained cells	92k	105k	68k
Major comorb.	obesity	various	cardiovascular
Time from Dx	2±1 days	3±3	N/A
Time from Sx	N/A	7±5	14±3
Time to death	38±20	20±13	35±14

B

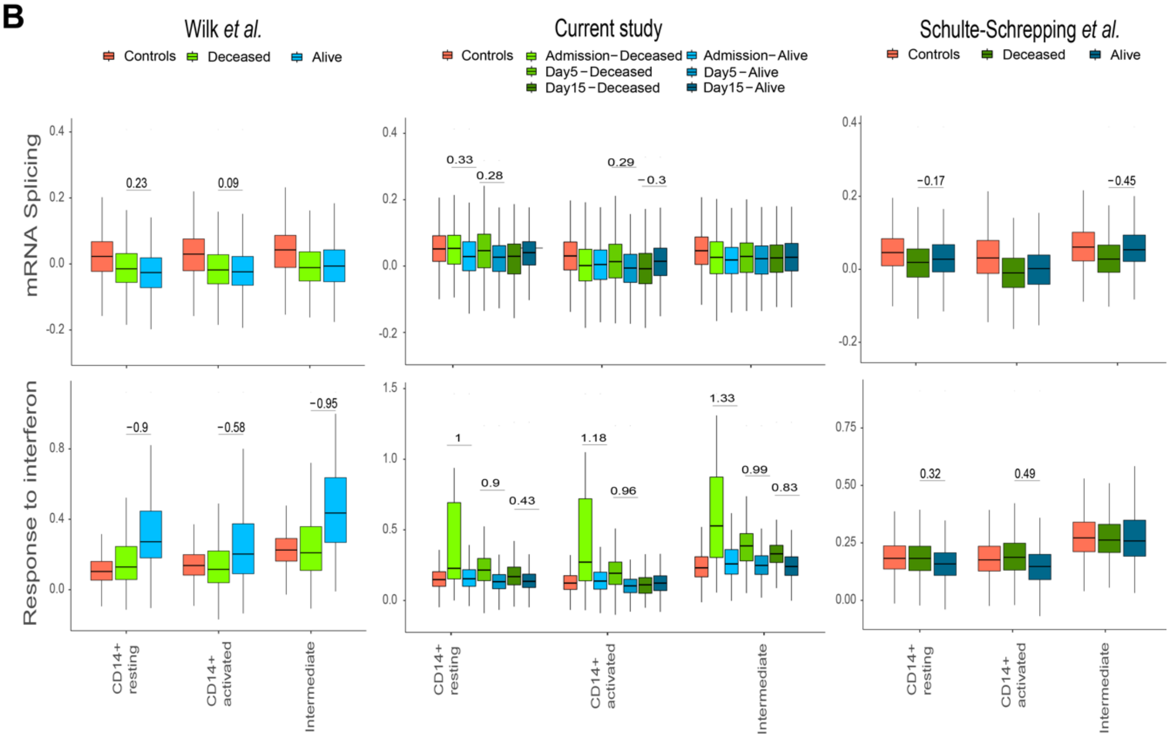

Figure S14. Analysis of three scRNAseq datasets

(A) Characteristics of the three data sets. **(B)** Module Scores for Response to Interferon and mRNA Splicing pathways in independent datasets. Module Score representing the overall transcriptional expression level in subtypes of CD14+ Monocytes for mRNA Splicing and Response to Interferon are show for three single cell datasets. Sample groups are coloured according to the key on top of the graph. The significance of the gene expression differences was tested by comparing the corresponding Module Score distribution in every group of cells using a wilcoxon rank-test approach (q-value <= 0.05). To provide a sample-size free evaluation of the difference in expression, Cohen's d effect size estimation is shown above each significant variation observed.

A

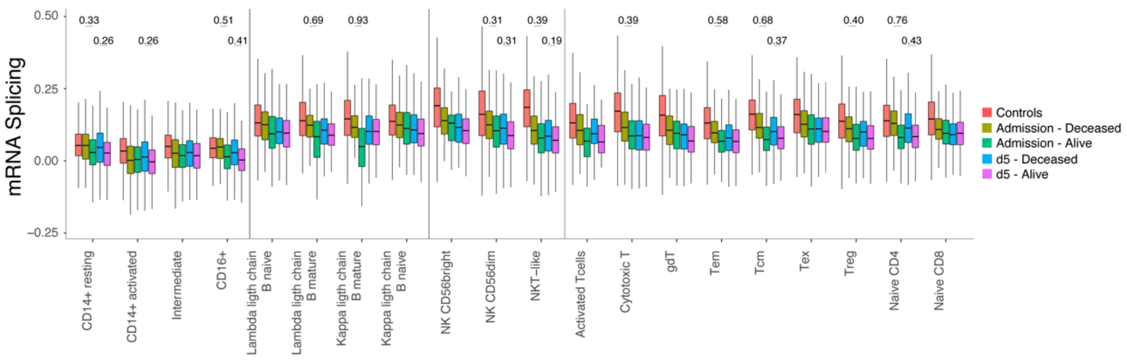

B

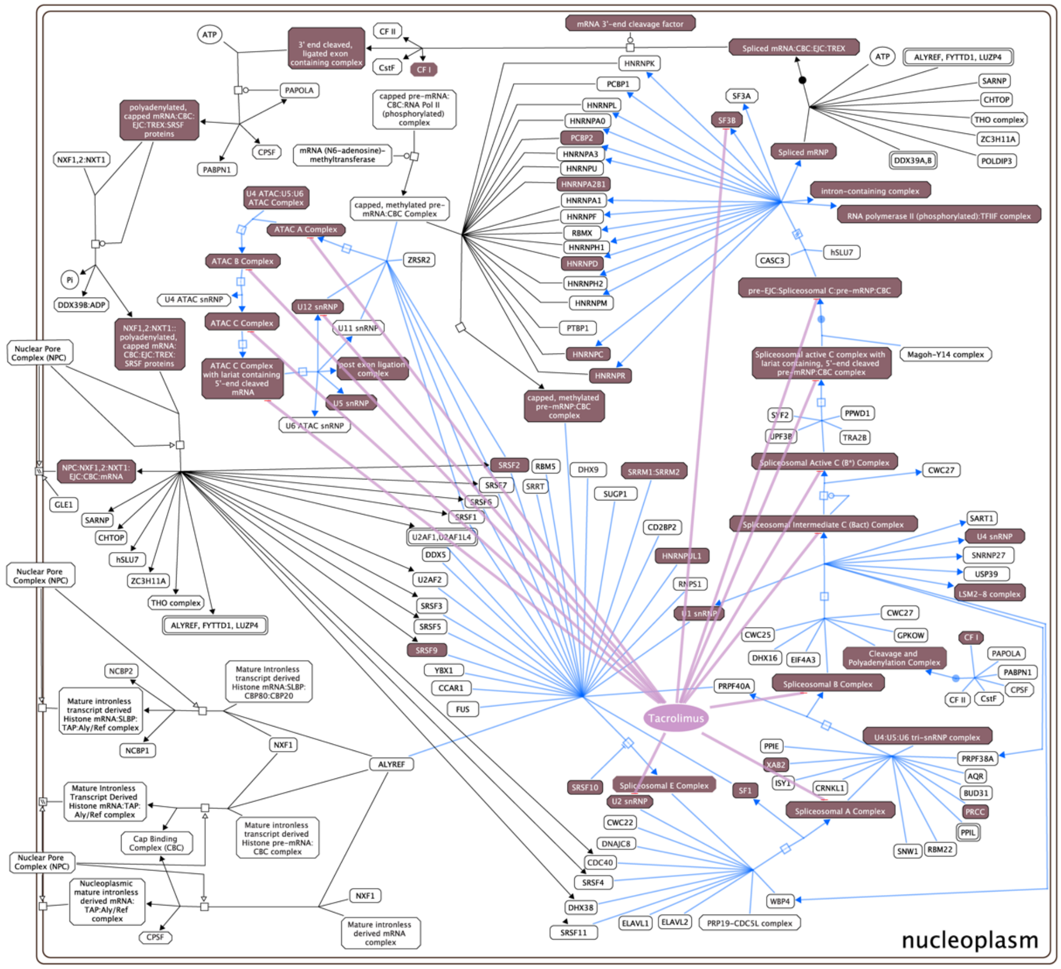

Figure S15: mRNA splicing pathway is elevated in multiple cell types.

(A) Evolution of the transcriptional expression for the *mRNA Splicing.* Differentially expressed *genes enriched in the* Reactome's *mRNA Splicing* pathway were used to generate a Module Score representing

the overall transcriptional expression level for the cell lineage indicated at the bottom of the graph. Module scores shown were derived at the D0 and D5 time-points. For each time point, the Module Score was estimated for the "Deceased" and "Alive" severely ill COVID-19 patients separately. Control samples from healthy subjects were included as reference for transcriptional expression levels. Significance of differences in the transcriptional expression levels between "Deceased" and "Alive" severely ill COVID-19 patients was tested by comparing the corresponding Module Score distribution in every group of cell subsets using a Wilcoxon rank-test approach (q-value <= 0.05). To provide a sample-size free evaluation of the difference in expression, Cohen's d effect size estimation is shown for every significant variation observed. **(B)** Tacrolimus interacts with mRNA splicing hubs identified by significant transcriptomic and epigenetic changes between patients at admission who will succumb to COVID-19 and those who will recover. The mRNA splicing portion of the Processing of Capped Intron-Containing Pre-mRNA pathway is indicated by light blue lines. Genes and hubs tagged by both transcriptomic and epigenetic changes are highlighted by a brown shade.

LIST OF SUPPLEMENTARY TABLES

Table S1. Differential gene expression analysis for each immune cell subpopulation identified by scRNAseq.

Table S2. Gene ontology and pathway enrichment analysis for differentially expressed genes per sub-population.

Table S3. Differential accessible chromatin analysis in monocytes of hospitalized COVID-19 patients.

Table S4. Gene ontology and pathway enrichment analysis for differentially accessible chromatin in monocytes.

Table S5. List of differentially methylated regions in monocytes of hospitalized COVID-19 patients at the admission.

Table S6. List of differentially methylated regions in monocytes of hospitalized COVID-19 patients at the follow-up.

Table S7. List of genes included in the Modules Scores.

Table S8. WGBS sequencing coverage and SNP removal statistics.

Table S9. Intersection of gene ontology and pathway enrichment analyses.
